# Lipocalin-2 Expressed in Innate Immune Cells Is an Endogenous Inhibitor of Inflammation in Murine Nephrotoxic Serum Nephritis

**DOI:** 10.1371/journal.pone.0067693

**Published:** 2013-07-04

**Authors:** Kathrin Eller, Andrea Schroll, Miriam Banas, Alexander H. Kirsch, Julia M. Huber, Manfred Nairz, Sergej Skvortsov, Günter Weiss, Alexander R. Rosenkranz, Igor Theurl

**Affiliations:** 1 Clinical Division of Nephrology, Department of Internal Medicine, Medical University of Graz, Graz, Austria; 2 Clinical Immunology and Infectious Diseases, Department of Internal Medicine VI, Innsbruck Medical University, Innsbruck, Austria; 3 Department of Nephrology, Internal Medicine II, University Hospital Regensburg, Regensburg, Germany; 4 Laboratory for Molecular Cell Biology, Internal Medicine I, Innsbruck Medical University, Innsbruck, Austria; 5 Department of Therapeutic Radiology and Oncology, Innsbruck Medical University, Innsbruck, Austria; French National Centre for Scientific Research, France

## Abstract

Lipocalin-2 (Lcn-2) is involved in divergent processes such as acute kidney injury or bacterial host defence. Our study was designed to evaluate the functional role of Lcn-2 in nephrotoxic serum nephritis (NTS). Since Lcn-2 is expressed in tubular epithelial cells as well as in cells of innate immunity such as macrophages and polymorphonuclear neutrophils (PMN), we induced NTS in wild-type (WT), Lcn-2 knock-out (KO) mice and WT/Lcn-2 KO chimeras. Mice lacking Lcn-2 exhibited more glomerular damage with increased proteinuria and interstitial leukocyte accumulation compared to WT mice. Chimeras able to express Lcn-2 in macrophages and PMN but not in epithelial cells were found to develop NTS comparable to wild-type controls. In contrast, chimeras expressing Lcn-2 in tubular epithelial cells with no expression in innate immune cells developed increased NTS due to decreased concerted apoptosis but increased necrosis and formation of damage-associated molecular patterns (DAMPs) such as high-mobility group box 1 (HMGB-1) in the kidney. *In vivo* blockade of HMGB-1, a toll-like receptor (TLR)-2 agonist, significantly reduced inflammation and NTS in Lcn-2 knock-out mice. In parallel, TLR-2 signalling was found to drive Lcn-2 transcription *in vitro*. Taken together, Lcn-2 expressed in innate immune cells is protective in NTS by inducing concerted apoptosis and inhibiting the formation of HMGB-1 thereby limiting cytokine production via TLR-2 signalling. In parallel, TLR-2 dependent transcription of Lcn-2 is an endogenous inhibitor of inflammation in NTS.

## Introduction

Lipocalin-2 (Lcn-2), also named Neutrophil-gelatinase associated lipocalin (NGAL) and 24p3, is a new marker in acute kidney injury as well as chronic kidney disease [1], [2]. It is a 21 kDa protein that is a member of the lipocalin superfamily and is expressed by renal tubular cells, hepatocytes and cells of the innate immune system such as polymorphonuclear neutrophils (PMN) and macrophages [3]–[5]. Lcn-2 gained first interest because of its bacteriostatic properties. It has the ability to bind the bacterial siderophores enterochalin, parabactin and carboxymycobactin, which are produced by bacteria as an iron delivery and acquisition system [6], [7]. In line, Lcn-2 knock-out (KO) mice have a pronounced defect in the defence against Gram-negative bacteria [7]–[9]. Furthermore, Lcn-2 has not only bacteriostatic effects, but has also been linked to cell apoptosis and proliferation [7], [8], [10], [11]. Even though Lcn-2 achieved attention as a very useful marker in acute and chronic renal failure [12], its function in kidney disease revealed elusive until very recently. Viau and co-workers evaluated the function of Lcn-2 in the progression of kidney disease in two experimental in vivo models of polycystic kidney disease. They found Lcn-2 to be actively involved in the pathogenesis of disease since Lcn-2 KO mice displayed significantly decreased disease progression as compared to wild-type (WT) mice. Mechanistically, they found Lcn-2 to act as a growth mediator via induction of EGFR signalling [13]. In renal ischemia reperfusion injury (IRI) the role of Lcn-2 is still obscure as effects of Lcn-2 administration in mice suffering from experimental renal ischemia could not be reproduced in Lcn-2 KO mice [8], [14]. The role of Lcn-2 in inflammatory driven kidney diseases such as nephrotoxic serum nephritis (NTS) is so far unclear. Recently, we have shown that Lcn-2 is up regulated in serum and urine of mice subjected to NTS [15]. NTS is a rapid progressive disease that is induced by the injection of a rabbit anti-mouse glomerular basement membrane (GBM) antibody and accelerated by a preceding immunization against rabbit IgG. Animals with NTS present with proteinuria, proliferative and inflammatory glomerular changes including crescent formation and leukocyte infiltrates, which are mainly located in the periglomerular and interstitial region [16], [17]. It has been proven to be dependent on Th17-activated PMN as well as Th1-activated macrophages [18]–[20], which both express Lcn-2 [3]–[5]. To further elucidate the role of Lcn-2 in NTS, we induced NTS in Lcn-2 KO and WT mice. In addition we generated chimeras by reconstituting lethally irradiated WT and Lcn-2 KO mice with WT and Lcn-2 deficient bone marrow cells, respectively. Interestingly, Lcn-2 KO mice and WT mice reconstituted with Lcn-2 deficient bone marrow cells developed significantly increased disease activity as compared to WT mice. PMN and macrophages lacking Lcn-2 did not undergo concerted apoptosis subsequently resulting in increased production of intracellular damage-associated molecular patterns (DAMPs), which increased disease activity via Toll-like receptor (TLR)-2 signalling. In parallel, Lcn-2 was increasingly transcribed by TLR-2 signalling possibly providing a rescue mechanism in NTS.

## Materials and Methods

### Ethics Statement

The protocol of animal experiments was approved by the Committee on the Ethics of Animal Experiments of the Austrian Ministry (GZ 66.011/0.111-11/10b/2008). All efforts were made to minimize suffering.

### Induction of Accelerated Nephrotoxic Serum Nephritis (NTS)

WT and Lcn-2 KO littermates were bred in the animal facility of the Medical University Innsbruck under SPF conditions. Lcn-2KO mice generated by Dr. Shizuo Akira (Japan) were obtained via Drs Alan Niels Borregaard, and Jack B. Cowland.

Eight to 10 week old female animals were used in all studies. Accelerated NTS was induced as described previously [21]. In brief, mice were pre-immunized subcutaneously with 100 µl of 2 mg/ml rabbit IgG (Jackson ImmunoResearch Laboratories Inc., West Grove, PA, USA) dissolved in incomplete Freund’s adjuvant (Sigma, St. Louis, MI, USA) and non-viable desiccated Mycobacterium tuberculosis H37a (Difco Laboratories, Detroit, MI, USA). After 3 days, heat-inactivated rabbit anti-mouse GBM antiserum was injected via the tail vein. One group of mice was treated with 2 mg/kg body weight chicken anti-mouse HMGB-1 antibody (Shino-Test Corporation, Tokyo, Japan) injected every second day intraperitoneally starting on the day before antiserum injection. Controls received an isotype control (Shino-Test Corporation). Chimeric mice were generated as described previously [21]. Shortly, bone marrow transplantation was undertaken using C57Bl/6 wild-type and Lcn-2 KO mice as donors and/or recipients. Bone marrow was isolated from respective donor mice. Recipient mice were irradiated with 1,200 rad from a caesium source to destroy their own bone marrow cells, and ∼10^7^ cells of donor bone marrow were injected into the tail vein. Induction of NTS was performed 4 weeks after bone marrow transplantation.

### Analysis of Peripheral Blood

Whole blood cell count was analyzed on a Vet-ABC Animal blood counter (Scil Animal Care Company).

### Detection of Urinary Albumin and Creatinine

Urinary albumin was determined by a double-sandwich ELISA (Abcam, Cambridge, MA, USA) as reported previously [21]. Urinary creatinine was quantitated spectrophotometrically using a picric acid-based method (Sigma, St. Louis, MI, USA).

### Detection of Urinary and Serum Lcn-2

Lcn-2 was evaluated in the urine and serum by using a commercially available evaluation kit (R&D Systems, Minneapolis, MN, USA).

### Histo- and Immunomorphological Evaluation of Renal Pathology

Formalin-fixed renal tissue was embedded in paraffin and cut in 4 µm sections. Renal sections were stained with periodic acid Schiff’s (PAS). In all cases a minimum of 50 equatorial glomerular cross sections were evaluated as previously described [22]. For evaluation of tubular necrosis, renal sections were stained with H&E and the number of tubular casts were evaluated in 6 low power fields.

PMN were stained with chloroacetate esterase (CAE) as described recently [17]. The positive cells in 6 Hpf were evaluated in a blinded fashion.

The three-layer immunoperoxidase staining of frozen tissue 4 µm sections was used for the detection of macrophages and T cell subpopulations in the kidney [21]. Macrophages were stained with rat anti-mouse anti-CD68 mAb (clone FA11 from Serotec, Oxford, UK). For the detection of CD4^+^, CD8^+^ T cells (clone YTS191.1 and clone KT15, both from Serotec), and Ly6G/Gr-1^+^ PMN (clone NIMP-R14 from Abcam) we used respective rat anti-mouse mAbs. Infiltration by CD8^+^ T cells, CD68^+^ macrophages, and Ly6G/Gr-1^+^ PMN was assessed quantitatively. For that purpose slides were scanned using an Aperio ScanScope (Aperio, Vista, CA, USA). We then used the Aperio ImageScope software 11.1.2.760 and the positive pixel algorithm (Version 2004-08-11; hue value: 0.1; hue width: 0.56; color saturation threshold 0.1) to quantify the sum of positive pixels in 6 analysis fields (Af, [squares of 2000×2000 pixels, equivalent to 6 550×550 µm squares]). In the case of CD4^+^ T cells and active Caspase 3, low-grade false positive background staining interfered with automated assessment and required visual identification of positive cells under the microscope. Therefore, cell quantitation was performed by counting the number of positive cells in 6 adjacent high-power fields (Hpf) of renal cortex and medulla. Samples were blinded before evaluation.

Lcn-2, PCNA, and active caspase 3 were stained on paraffin sections using the three-layer immunoperoxidase staining protocol subsequent to standardized heat-mediated antigen retrieval with an automated de-cloaking chamber (Biocare Medical, Concord, CA, USA). Tissue sections were then stained with a goat anti-mouse Lcn-2 antibody (R&D Systems), a mouse anti-mouse PCNA antibody (BioLegend, San Diego, CA, USA), or a rabbit anti-mouse active Caspase 3 antibody (Abcam). PCNA staining was performed using the M.O.M. staining kit (Vector Labs, Burlingame, CA, USA). For double staining of Gr-1, CD-68 and Lcn-2, slides were first stained for Gr-1 or CD68, respectively, using the ImmPACT SG substrate kit (Vector Labs) followed by staining for Lcn-2 using 3-amino-9-ethyl-carbazole as a substrate.

For doublestaining of Mac-2 (Cederlane, Burlington, USA, # CL8942AP) and TLR2 (ProSci, Poway, USA, # XW-7622) respective antibodies were used and applied as previously described [23].

### TUNEL-staining of Kidney Sections

Terminal transferase dUTP nick end labelling (TUNEL) was performed using a commercially available staining kit (Roche, Basel, Switzerland) with the following changes to the recommended protocol. Glass slides were pre-treated with Vectabond (Vector Laboratories, Burlingame, CA, USA) according to the manufacturer’s protocol. After rehydratation tissue sections were treated with 10 µg/ml Proteinase K (Roche) and TUNEL mixture was applied onto each section. The slides were incubated in a humidified chamber for 60 min at 37°C in the dark. Finally, slides were rinsed 3 times in PBS. Positive controls were generated by incubating samples from control kidneys at RT for 10 min with 100 µl of a 1000 U/ml solution of DNase I (Invitrogen, Carlsbad, CA, USA) in DNase I buffer (50 mM Tris-HCl, pH 7.5, 1 mg/ml BSA), in order to induce strand breaks. Sections were then washed 3 times in PBS. Negative controls were created by incubating sections with label solution alone. Nuclei were counterstained with 4′, 6-Diamidino-2-phenylindol, dihydochloride (DAPI) (Molecular Probes Europe BV, Leiden, The Netherlands) at a concentration of 300 nM. TUNEL positive cells in 6 Hpf were quantified by a blinded observer.

### Measurement of HMGB1 by Western Blotting

Kidney samples were homogenized and lysed on ice by using Triton lysis buffer (37.6 mM KCl, 24.8 mM Tris base, 1% Triton X-100) supplemented with 1% protease inhibitor cocktail (Sigma). Five µg of each sample were analysed on 8% SDS-PAGE and blotted onto a hydrophobic polyvinylidene difluoride membrane (Amersham Biosciences Corp, Piscataway, NJ, USA). Rabbit anti-HMGB1 antibody (Abcam, Cambridge, UK) was used at a final concentration of 0.5 µg/ml. As a secondary antibody HRP-conjugated goat anti-rabbit IgG (Dako, Glostrup, Denmark) at a dilution of 1∶2000 was used. After chemiluminescent reaction using an enhanced chemiluminescence (ECL) Western blot reagent (Amersham Biosciences Corp), the blots were exposed to HyperfilmTM ECL (Amersham Biosciences Corp). After stripping of the Western blots using Mild Stripping solution (Millipore, Billerica, MA, USA) according to the Manufacturer’s protocol blots were incubated with mouse anti-beta actin antibody (clone AC-74, Sigma) at a final concentration of 1 µg/ml. As a secondary antibody HRP-conjugated goat anti-mouse IgG (Dako) at a dilution of 1∶2000 was used. Detection was performed as described above.

### Detection of Circulating Mouse Anti-rabbit IgG

For detection of circulating mouse anti-rabbit IgG 96-well plates (Greiner, Kremsmuenster, Austria) were coated with 100 µg/ml rabbit IgG (Jackson ImmunoResearch Laboratories Inc.) in carbonate/bicarbonate buffer (pH 9.5). After blocking with 1% BSA plates were incubated with serial-doubling dilutions of mouse serum. Bound mouse IgG was detected by HRP-conjugated goat-anti-mouse IgG (Dako, Glostrup, Denmark).

### Reverse Transcription (RT) Real-time Polymerase Chain Reaction (PCR)

Total RNA was isolated using TRIzol® (Sigma) according to a standard protocol. Thereafter, 2 µg of total RNA was reverse transcribed using Superscript III Transcription Kit (Invitrogen) and random primers (Roche, Basel, Switzerland). Real-time PCR was performed on an ABI Prism 7700 (Applied Biosystems, Foster City, CA, USA) or a CFX96 Real-Time System (Biorad, Hercules, CA, USA). The following TaqMan PCR primers and probes were used for evaluation of the *in vivo* experiments: *TNF-alpha:* 5′-TTCTATGGCCCAGACCCTA-3′, 5′- TTGCTACGACGTGGGCTACA-3′, FAM-CTCAGATCATCTTCTCAAAATTCGAGTGACAAGC-BHQ1, *IL-6∶*5′- TGTTCTCTGGGAAATCGTGGA-3′, 5′-AAGTGCATCATCGTTGTTCATACA-3′, FAM- ATGAGAAAAGAGTTGTGCAATGGCAATTCTG- BHQ1, *t-bet:* 5′- CCTGTTGTGGTCCAAGTTCAAC-3′, 5′- CACAAACATCCTGTAATCGCTTGT-3′, FAM- ATCATCACTAAGCAAGGACGGCGAATGTTCC-BHQ1, *IFNγ:* 5′- TCAAGTGGCATAGATGTGGAAGAA-3′, 5′- TGGCTCTGCAGGATTTTCATG-3′, FAM- TCACCATCCTTTTGCCAGTTCCTCCAG-BHQ1, *IL-17A:* 5′- GCTCCAGAAGGCCCTCAG-3′, 5′- CTTTCCCTCCGCATTGACA-3′, FAM- ACCTCAACCGTTCCACGTCACCCTG-BHQ1, *RORγt:* 5′- CCGCTGAGAGGGCTTCAC-3′, 5′- TGCAGGAGTAGGCCACATTACA-3′, FAM- AAGGGCTTCTTCCGCCGCAGCCAGCAG-BHQ1, *GATA-3∶*5′- CTACCGGGTTCGGATGTAAGTC-3′, 5′- GTTCACACACTCCCTGCCTTCT-3′, FAM- AGGCCCAAGGCACGATCCAGC-BHQ1, *HPRT:* 5′- GACCGGTCCCGTCATGC-3′, 5′- TCATAACCTGGTTCATCATCGC-3′, FAM- ACCCGCAGTCCCAGCGTCGTC-BHQ1. HPRT-1 was used as a reference gene. Sequences of TLR and Lipocalin primers for real-time PCR evaluations in cell culture experiments are shown in Table 1.

**Table 1:** Sequences of PCR primers used for real-time PCR of cell culture experiments.

Gene of interest	Forward Primer	Reverse Primer
**TLR1**	5′-ggacctacccttgcaaacaa-3	5′-ggtggcacaagatcaccttt-3
**TLR2**	5′-ttatcttgcgcagtttgcag-3	5′-ctcccacttcaggctctttg-3
**TLR3**	5′-agcatcaaaagaagccgaaa-3	5′-cttgctgaactgcgtgatgt-3
**TLR4**	5′-gctcctggctaggactctga-3	5′-tgtcatcagggactttgctg-3
**TLR5**	5′-ctggggacccagtatgctaa-3	5′-acagccgaagttccaagaga-3
**TLR6**	5′-acacaatcggttgcaaaaca-3	5′-ggaaagtcagcttcgtcagg-3
**TLR7**	5′-attccttgcctcctgaggtt-3	5′-gctgaggtccaaaatttcca-3
**TLR8**	5′-agtttgcacattccctggac-3	5′-agaggaagccagagggtagg-3
**TLR9**	5′-actgagcaccctgcttcta-3	5′-ggctcaggctaagacactgg-3
**Lcn-2**	5′-gcctcaaggacgacaacatca-3	5′-caccacccattcagttgtcaat-3
**IL6**	5′-cagaggataccactcccaaca-3	5′-ttctgcaagtgcatcatcgt-3
**TNF-α**	5′-cagttggtggtttgctacga-3	5′-ccccaaagggatgagaagtt-3

### Cell Culture Experiments

Immortalized mouse macrophages (RAW264.7) were obtained from the European Collection of Cell Culture. An immortalized murine renal distal convoluted tubular cell line was kindly provided by Tobias Bergler [24], [25]. Macrophages were grown in Dulbecco’s modified Eagle’s medium (DMEM) D6546 (Sigma Aldrich, Taufkirchen, Germany) supplemented with 10% bovine serum (PAA, Pasching, Austria), 2 mM glutamine (PAA) and 1% penicillin-streptomycin (PAA; 100 U/ml and 100 µg/ml) in an atmosphere of 95% air/5% CO_2_ at 37°C (100,000 cells per/well, cultured in six-well plates for 24 hours). Renal distal convoluted tubular cells were grown in DMEM Nutrient Mixture F-12 HAM D8473 (Sigma Aldrich) supplemented with 5% bovine serum and 1% penicillin-streptomycin in an atmosphere of 95% air/5% CO_2_ at 37°C. To test for functional responses, cultured macrophages and distal convoluted tubular cells were stimulated for 24 hours with 10 µg/ml lipoteichoic acid (LTA) extracted from Staphylococcus aureus (Invivogen, San Diego, USA) and with 10 µg/ml Peptidoglycan (PGN) prepared from E.coli 0111:B4 (Invivogen). Extraction of total RNA was performed using the RNeasy Mini Kit (Qiagen, Hilden, Germany) with additional DNase digestion. Subsequent real-time PCR was performed as described previously [23].

### Treatment of Macrophages and Distal Convoluted Tubular Cells with siRNA

Macrophages (100,000 cells per/well, cultured in six-well plates) were incubated with 5 nM TLR2 siRNA SI00204316 (Qiagen, Hilden, Germany ) for 24 h using HiPerfect Transfection Reagent (Qiagen, Germany) for transient transfection. Distal convoluted tubular cells were incubated with 10 nM TLR2 siRNA. AllStars Negative control (Qiagen) has been used as control siRNA. Fluorescence-labeled siRNA was used in pilot experiments to determine optimal transfection rates according to the manufacturer’s protocol.

### Apoptosis Assays

Apoptosis of murine macrophages was induced by staurosporin (Sigma Aldrich). A time course with different doses of staurosporin was peformed and for further experiments treatment with 500 ng/ml staurosporin for 6 hours was used. For analysis of Lcn-2 effects, cells were pretreated with Lcn-2 (R&D Systems, Wiesbaden, Germany #1857-LC-050; Cell Signalling, Frankfurt, Germany #8199) 0,5 µg/ml, 1 µg/ml and 10 µg/ml and Lcn-2 siRNA 5 mM (Qiagen, Hilden, Germany #SI01088381). Apoptosis was studied using two different methods: For visualization of chromatin fragmentation, murine macrophages were seeded on cell culture plates (Greiner BioOne, Bahlingen, Germany). After treatment with test substances cells were stained with NucBlue Live Cell Stain (Hoechst 33341, Life Technologies; Darmstadt, Germany). The percentage of apoptotic cells was determined by immunofluorescence microscopy, counting nuclei with condensed and fragmented chromatin. Three sets of experiments were performed and at least 300 cells were analyzed per condition. For measurement of caspase-3/7 activity a commercial assay (Promega, Mannheim, Germany #G6320) was used according to the manufacturer’s instructions. After induction of apoptosis as described above, caspase-3/7 specific proteolytic activity was measured with a luminometer (Infinite M200pro, Tecan, Maennedorf, Switzerland). Three sets of experiments were performed.

### Statistical Analysis

Data are presented as mean ± SEM. Normal distribution of the data was assessed by the Kolmogorov-Smirnov test with Lilliefors correction. Groups were compared by either non-parametric Mann-Whitney U test or unpaired t test as appropriate, depending on the distribution of the tested variable. A two-tailed p<0.05 was considered statistically significant. When comparing three groups we performed ANOVA or the Kruskal-Wallis test, respectively. When significances were detected, the different groups were compared by unpaired t test or non-parametric Mann-Whitney U test, respectively, using the Bonferroni method to adjust the significance level for multiple testing. All statistical analyses were done with SPSS 15.0 for Windows (SPSS, Chicago, IL, USA).

## Results

### Kidney Injury is Aggravated in Lcn-2 KO Mice after Induction of NTS

**Figure 1:**
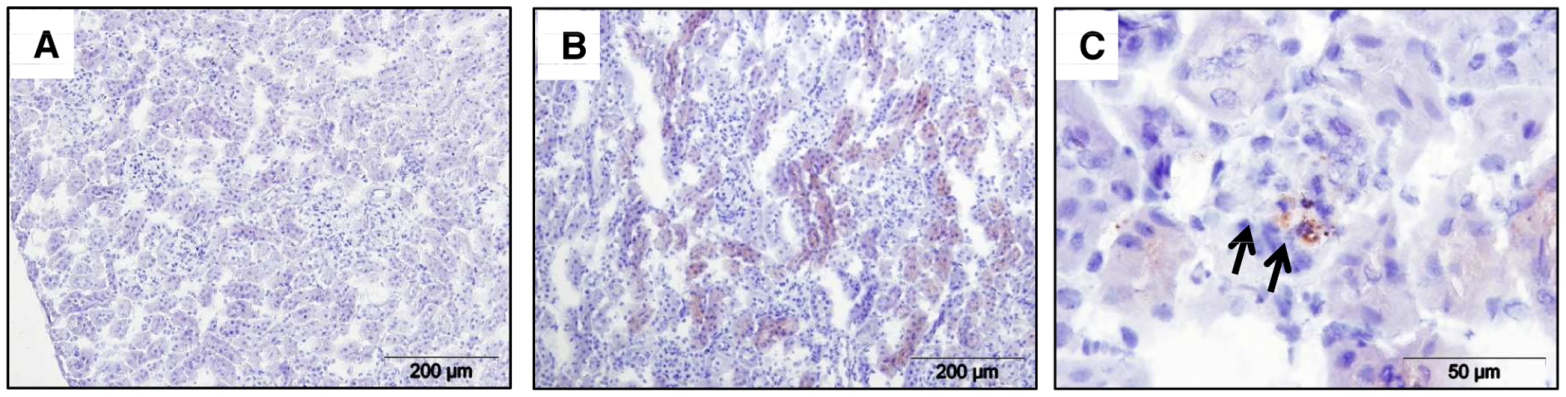
Lcn-2 is expressed in tubular epithelial and innate immune cells NTS. C57BL/6 mice were subjected to NTS and were followed for 7 days. Kidneys were stained for Lcn-2. (A) The isotype control is shown. (B+C) Representative pictures are presented. Black arrows indicate infiltrating immune cells stained positively for Lcn-2.

**Figure 2:**
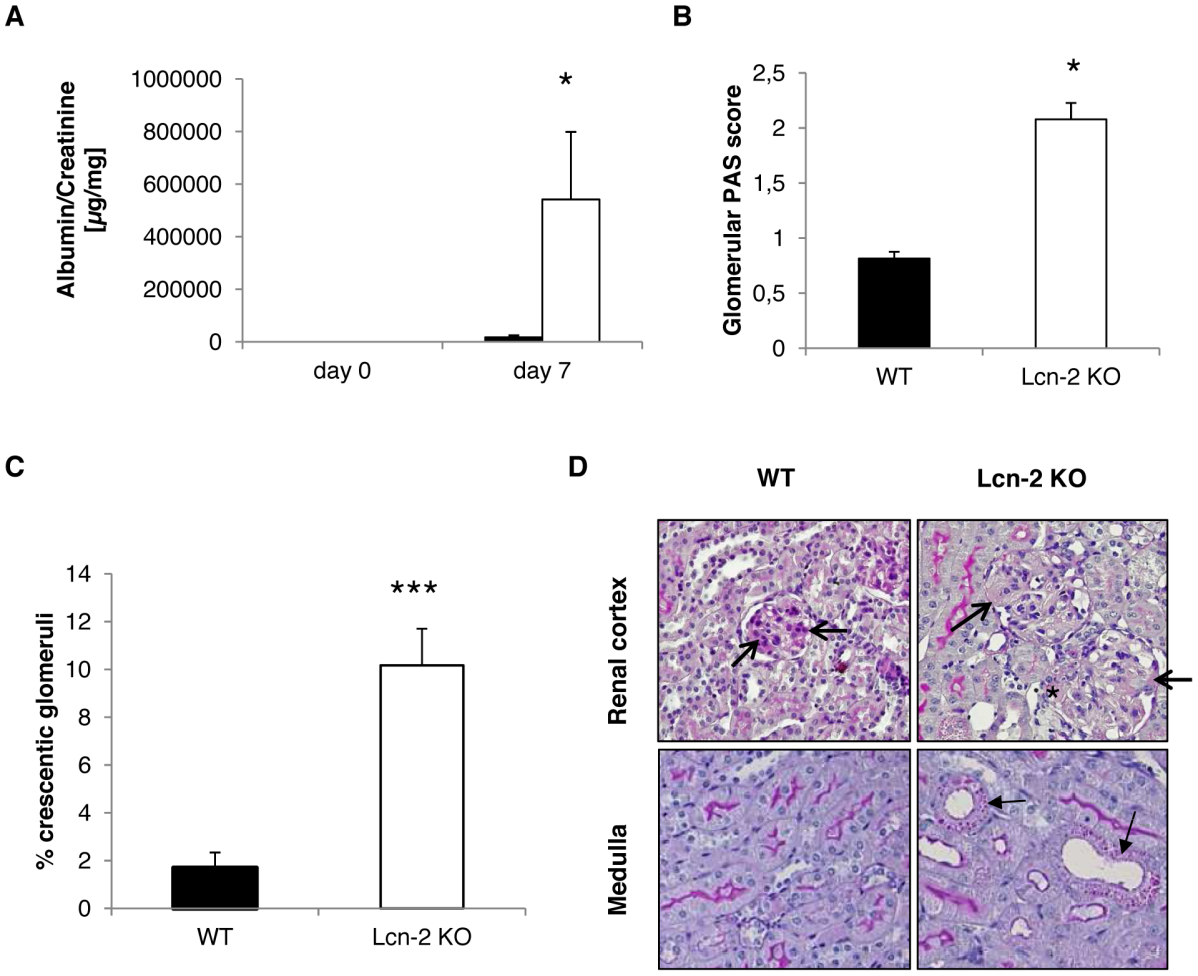
Lcn-2 KO mice are increasingly susceptible to NTS. **WT (black bar; n = 13) and Lcn-2 KO mice (white bar; n = 12) were subjected to NTS.** (A) Albuminuria was evaluated on day 0 and day 7 after NTS induction. (B) On day 7 after NTS induction kidney sections were evaluated for PAS positive deposits. (C) The percentage of crescentic glomeruli was quantified in WT and Lcn-2 KO mice. Data are given as means ± SEM. *p<0.05. (D) Representative PAS-stained sections of WT and Lcn-2 KO mice are shown. PAS positive glomeruli are marked by open arrows. A small crescent formation is marked by an asterix. Tubular cells with PAS positive material inserted because of heavy proteinuria are marked by closed arrows. Magnification x400.

Lcn-2 was significantly up-regulated in the serum and urine of mice during the course of NTS [15]. We detected Lcn-2 protein expression 7 days after induction of NTS mainly in proximal tubular cells, but also in infiltrating immune cells in the kidney (Figure 1A–C and Figure S1). The immune cells were found to co-express the neutrophil marker Gr-1 or the macrophage marker CD68 and Lcn-2 (Figure S1). To further evaluate the role of Lcn-2 in NTS, we induced NTS in Lcn-2 KO and WT mice. Lcn-2 KO mice displayed significantly increased albuminuria on day 7 after NTS induction as compared to WT controls (Figure 2A). Mild hyper-cellularity and focal deposition of PAS positive material were detected in glomeruli of WT controls 7 days after induction of NTS (Figure 2D). This kidney pathology was found to be more pronounced in Lcn-2 KO mice (Figure 2D). Quantification of PAS-positive deposits in glomeruli (Figure 2B) as well as percentage of crescent formation (Figure 2C) revealed a significant increase in the Lcn-2 KO as compared to WT mice. Whereas WT mice did not show a tubular pathology, tubular cells of Lcn-2 KO mice were found to have PAS-positive inserts reflecting reabsorbed protein due to extensive proteinuria (Figure 2D). Of note, no difference in the autologous antibody production between the two groups was detected ruling out a role of B cells in this disease model (Figure S2).

### Increased Cellular Kidney Infiltration in Lcn-2 KO Mice Subjected to NTS

**Figure 3:**
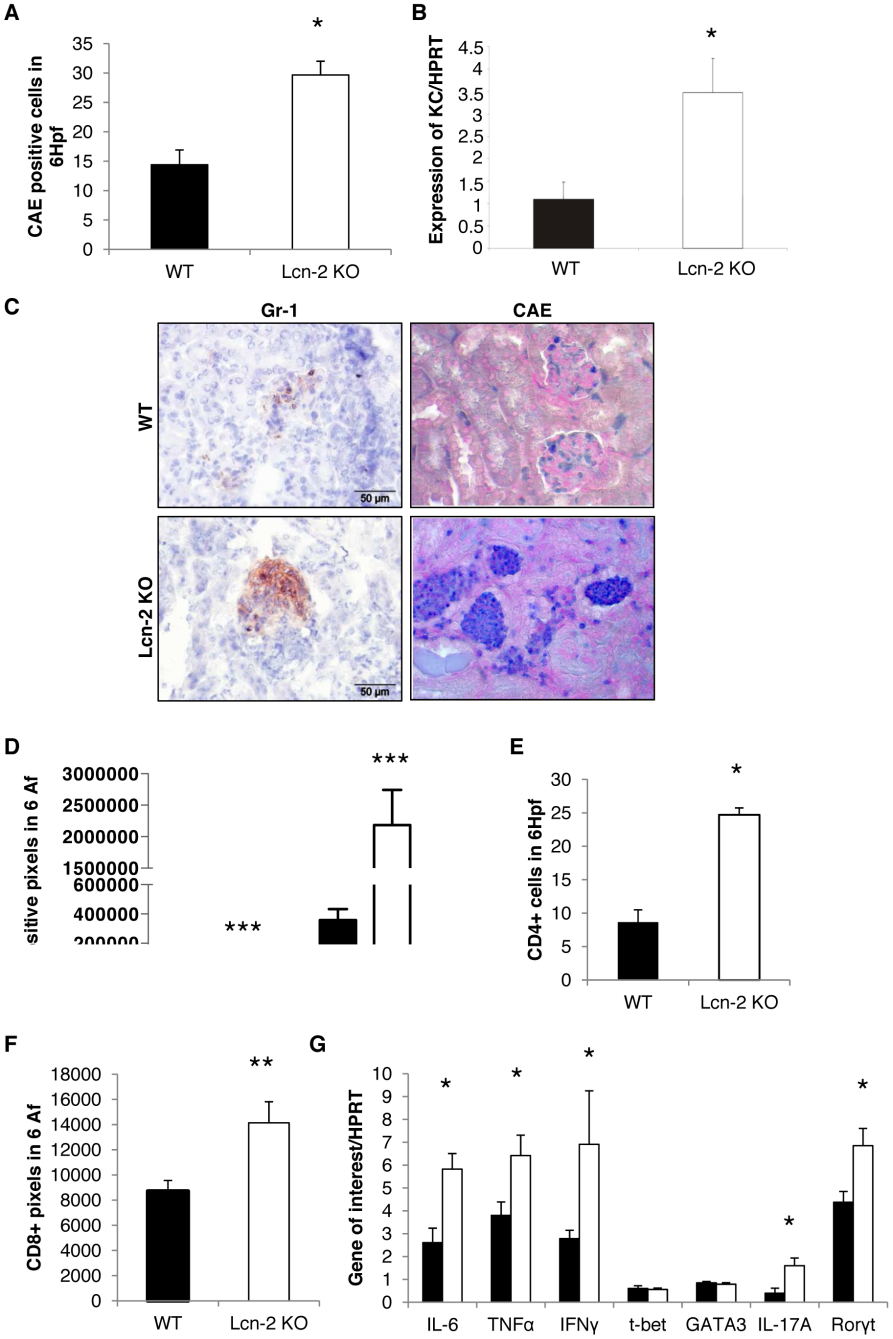
Lcn-2 KO mice display increased inflammatory cell infiltration of kidneys after NTS. WT (black bar; n = 13) and Lcn-2 KO mice (white bar; n = 12) were subjected to NTS. (A) Seven days after induction of NTS kidney sections were evaluated for the infiltration of CAE^+^ cells. The number of positive cells in 6 high power fields is given. (B) cDNA isolated from kidney samples was analysed for the KC expression. The relative abundance of KC to the house-keeping gene HPRT is provided. (C) Representative pictures of kidneys stained for Gr-1^+^ (left column) and CAE^+^ (right column) cells are given. The CAE^+^ cells are stained in dark blue, the Gr-1^+^ cells in brown. Kidney sections were analyzed for Gr-1 and CD68 (D), CD4 (E), and CD8 (F). (G) cDNA isolated from kidney samples was analyzed for the expression of the respective genes. The fold increase compared to healthy controls is provided. All data are given as mean ± SEM. Af = analysis field. Hpf = high power field. *p<0.05, **p<0.01, and ***p<0.001.

Significantly increased numbers of PMN were detected in Lcn-2 KO mice 7 days after NTS induction as shown by CAE and Gr-1 staining (Figure 3A, C, D) compared to WT mice. Whereas few PMN were found to infiltrate the glomeruli and were absent in the interstitium of WT mice, they massively infiltrated the interstitium, glomerulus and tubular lumen of Lcn-2 KO mice (Figure 3C). In line, increased mRNA expression of the PMN marker KC was detected in kidneys of Lcn-2 KO mice as compared to WT mice 7 days after induction of NTS (Figure 3B). Furthermore, significantly increased numbers of CD68^+^ macrophages (Figure 3D), CD4^+^ and CD8^+^ T cells (Figure 3E,F) were found in kidneys of Lcn-2 KO mice. Again, they mainly infiltrated the periglomerular and interstitial region of Lcn-2 KO kidneys. Only a few CD68^+^ macrophages and CD4^+^ T cells were detected within glomeruli (data not shown). Upon cytokine profiling of the kidneys, significantly increased mRNA expression of the monocyte/macrophage markers IL-6, TNF-α and IFN-γ was detected in Lcn-2 KO mice (Figure 3G). Furthermore, the TH17 marker IL-17A and RORγt were significantly increased in Lcn-2 KO mice (Figure 3G). No difference in the mRNA expression of the TH1 and TH2 master regulator t-bet and GATA3, respectively, was detected between the two groups (Figure 3G). Since immune-regulation in NTS has been shown to be of importance [16], [26]–[28], cytokine profiling in the lymph nodes was performed. Only IL-6 mRNA expression was significantly changed in Lcn-2 KO mice as compared to WT mice 7 days after NTS induction. All other cytokines evaluated did not differ between the two groups (Table 2). In peripheral blood, we detected significantly increased numbers of monocytes and PMN in Lcn-2 KO mice as compared to WT controls 7 days after NTS induction (Table 3).

**Table 2:** Cytokine expression in lymph nodes of WT and Lcn-2 KO mice 7 days after NTS induction.

Gene	WT	Lcn-2 KO	p
**IL-6**	4.03±4.57	1.11±0.80	0.04
**TNFα**	2.45±1.06	2.40±0.73	0.93
**IFNγ**	2.97±1.87	2.81±2.72	0.87
**t-bet**	2.60±0.58	3.01±0.94	0.22
**GATA3**	0.84±0.34	0.65±0.20	0.11
**Rorγt**	2.27±1.80	1.47±1.01	0.19
**IL-17A**	1.94±1.11	2.15±1.59	0.71

WT (n = 13) and Lcn-2 KO mice (n = 12) were subjected to NTS and followed for 7 days. cDNA isolated from lymph nodes was analyzed for the expression of the respective genes. The respective gene/HPRT ratio is given. Data are provided as mean ± SEM.

**Table 3:** Whole blood cell count in WT and Lcn-2 KO mice.

	WT	Lcn-2 KO	P
**Wbc**	9.49±3.42	9.77±2.32	0.830
**Rbc**	9.63±0.50	9.02±0.70	0.210
**Plt**	840.15±245.48	651.83±232.65	0.620
**Hgb**	13.43±0.46	12.61±1.03	0.023
**Hct**	48.78±1.70	45.26±3.59	0.008
**Lymphocytes**	6.15±2.48	6.07±2.60	0.938
**Monocytes**	0.62±0.34	0.97±0.34	0.022
**Neutrophils**	2.07±0.52	3.50±0.95	0.001
**Eosinophils**	0.14±0.05	0.53±0.86	0.164

WT (n = 13) and Lcn-2 KO mice (n = 12) were subjected to NTS and followed for 7 days. On day 7 whole blood was analyzed for the white (wbc) and red blood cell count (rbc), for the number of platelets (plt), the hemoglobin levels (hgb), the haematocrit (hct) as well as for the number of lymphocytes, monocytes, neutrophil and eosinophil granulocytes. Data are given as mean ± SEM.

### Apoptosis is Decreased whereas HMGB-1 is Increased in Lcn-2 KO after NTS Induction

**Figure 4:**
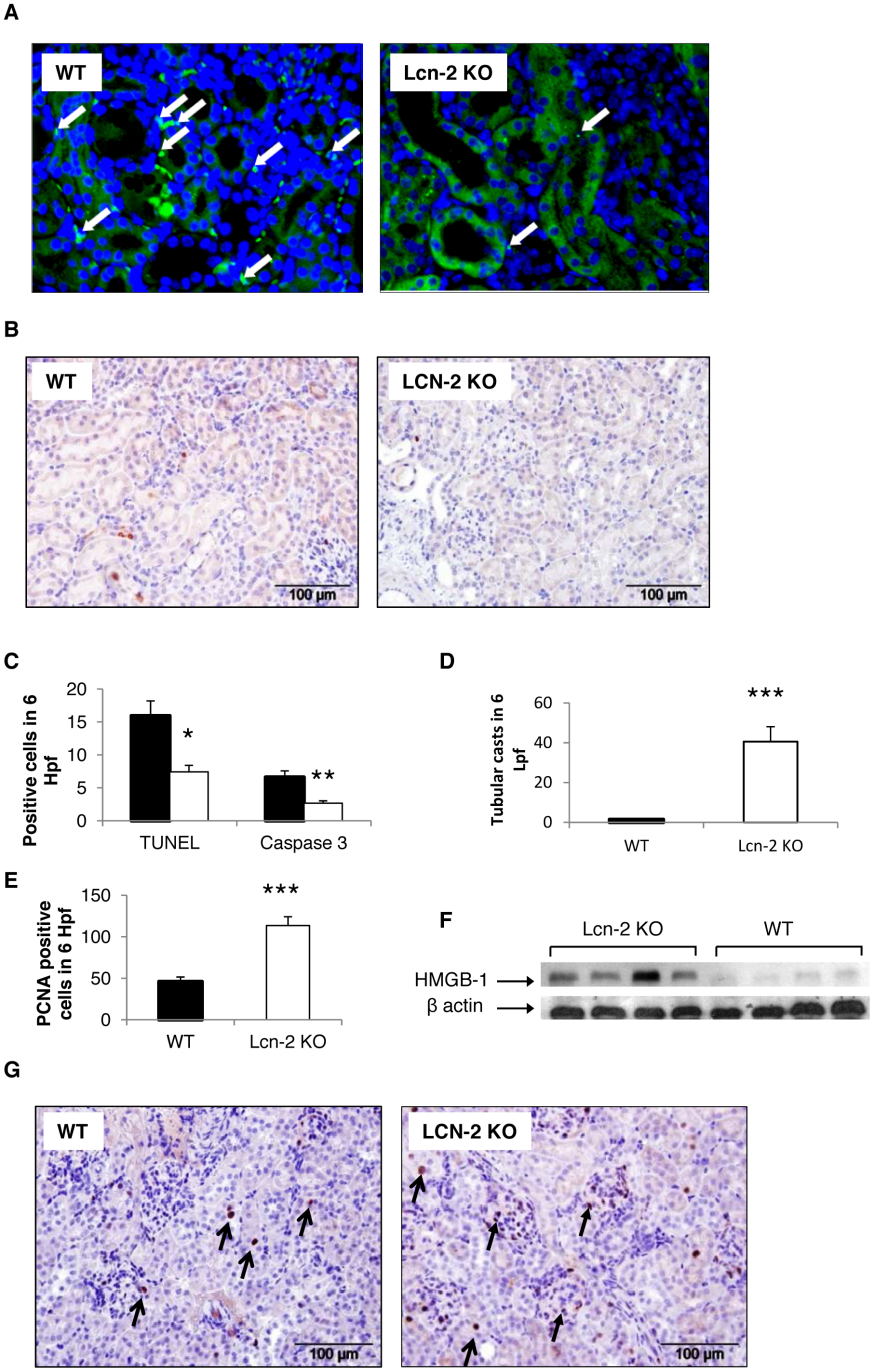
Lcn-2 KO show decreased apoptosis but increased proliferation in NTS. WT (black bar; n = 13) and Lcn-2 KO mice (white bar; n = 12) were subjected to NTS. Seven days after induction of NTS kidney sections were (A) TUNEL stained and stained for (B) active Caspase-3. Apoptotic cells are marked with arrows. (C) The number of TUNEL and active Caspase-3 positive cells was quantified. Furthermore, sections were (D) H&E stained and evaluated for tubular necrosis. (E+G) Additionally, kidneys of WT and Lcn-2 KO mice were evaluated for proliferating cells 7 days after NTS induction by performing PCNA stainings. (E) Quantification and (G) representative pictures are shown._Magnification x400. Open arrows show representative PCNA-positive tubular epithelial cells. Closed arrows reflect representative PCNA-positive infiltrating immune cells. (F) Protein isolated from kidneys of Lcn-2 KO and WT mice was analyzed for HMGB-1 by Western blotting. Beta-actin reblotting was performed as a loading control. One representative blot is shown. All data are given as means ± SEM. Hpf = high power field. Lpf = low power field. *p<0.05, **p<0.01 and ***p<0.001.

Lcn-2 has been proposed to induce concerted apoptosis [10], [11], but also to decrease proliferation [13], [29], which we evaluated by TUNEL and Caspase-3 staining and PCNA staining, respectively. As early as day one and four after NTS induction, decreased numbers of Caspase 3-positive apoptotic cells were detected in kidneys of Lcn-2 KO mice as compared to WT mice, but significance was not reached (Figure S3). On day seven after induction of NTS significantly decreased numbers of TUNEL- or Caspase 3-positive cells infiltrating kidneys of Lcn-2 KO mice were detected as compared to WT controls (Figure 4A–C). In contrast, we found increasing rates of tubular necrosis evaluated by H&E staining (Figure 4D) and proliferation detected by PCNA stainings (Figure 4E+G).

**Figure 5:**
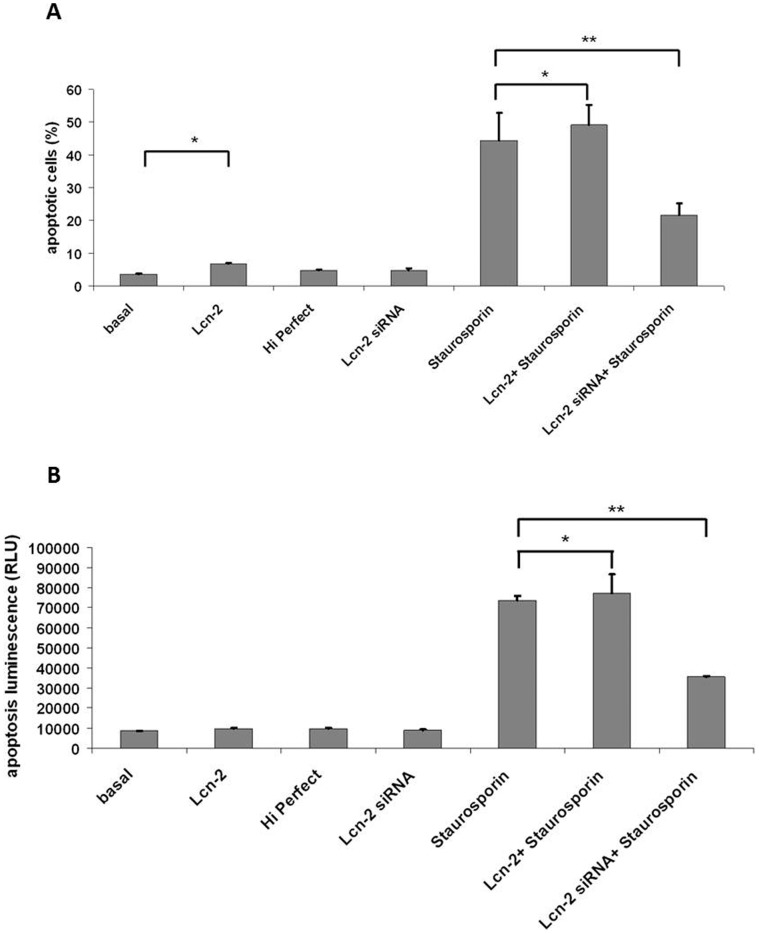
Effect of Lcn-2 and Lcn-2 siRNA on staurosporin-induced cell death of murine macrophages. (A) Percentages of apoptotic macrophages were determined after visualization of fragmented chromatin using Hoechst dye. (B) Caspase-3/7 activity was quantitated spectrophotometrically in macrophages. Lcn-2 incubation significantly increased number of apoptotic cells, prestimulation with Lcn-2 si-RNA prior to induction of cell death reduced macrophage apoptosis. Statistically significant differences are depicted: *,p<0.05; **,p<0.01.

To further evaluate the role of Lcn-2 in apoptosis we evaluated the *in vitro* effects of Lcn-2 and Lcn-2 siRNA on cultured murine macrophage survival in staurosporin-induced cell death by using two different methods. Visualization of fragmented chromatin was performed by Hoechst staining. Stimulation of macrophages with Lcn-2 increased staurosporin-induced cell death moderately but statistically significant. Prestimulation with Lcn-2 siRNA prior to induction of apoptosis with staurosporin markedly reduced apoptosis (Figure 5A). Induction of cell death of macrophages by staurosporin increased Caspase-3/7 activity approximately 8-fold compared with control conditions. Treatment of Lcn-2 preincubated cells with staurosporin increased caspase-3/7 activity, whereas preicubation with Lcn-2 siRNA significantly reduced the induced cell death (Figure 5B).

Since decreased concerted apoptosis leads to necrosis and the development of DAMPs [30], [31], we evaluated kidneys for the DAMP HMGB-1. A significantly increased expression of high-mobility group box 1 (HMGB-1) – an intracellular protein that can also activate the innate immune system – was found in Lcn-2 KO mice 7 days after NTS induction as compared to WT controls (Figure 4F). Of note, significantly increased numbers of proliferating cells detected by PCNA staining were found in the kidney of Lcn-2 KO mice 7 days after NTS induction (Figure 4E). These cells were on the one hand tubular epithelial cells, but also infiltrating interstitial immune cells (Figure 4G).

### Studies in Bone Marrow Chimeras - WT Mice Reconstituted with Lcn-2 KO Bone Marrow Cells are Increasingly Susceptible to NTS

**Figure 6:**
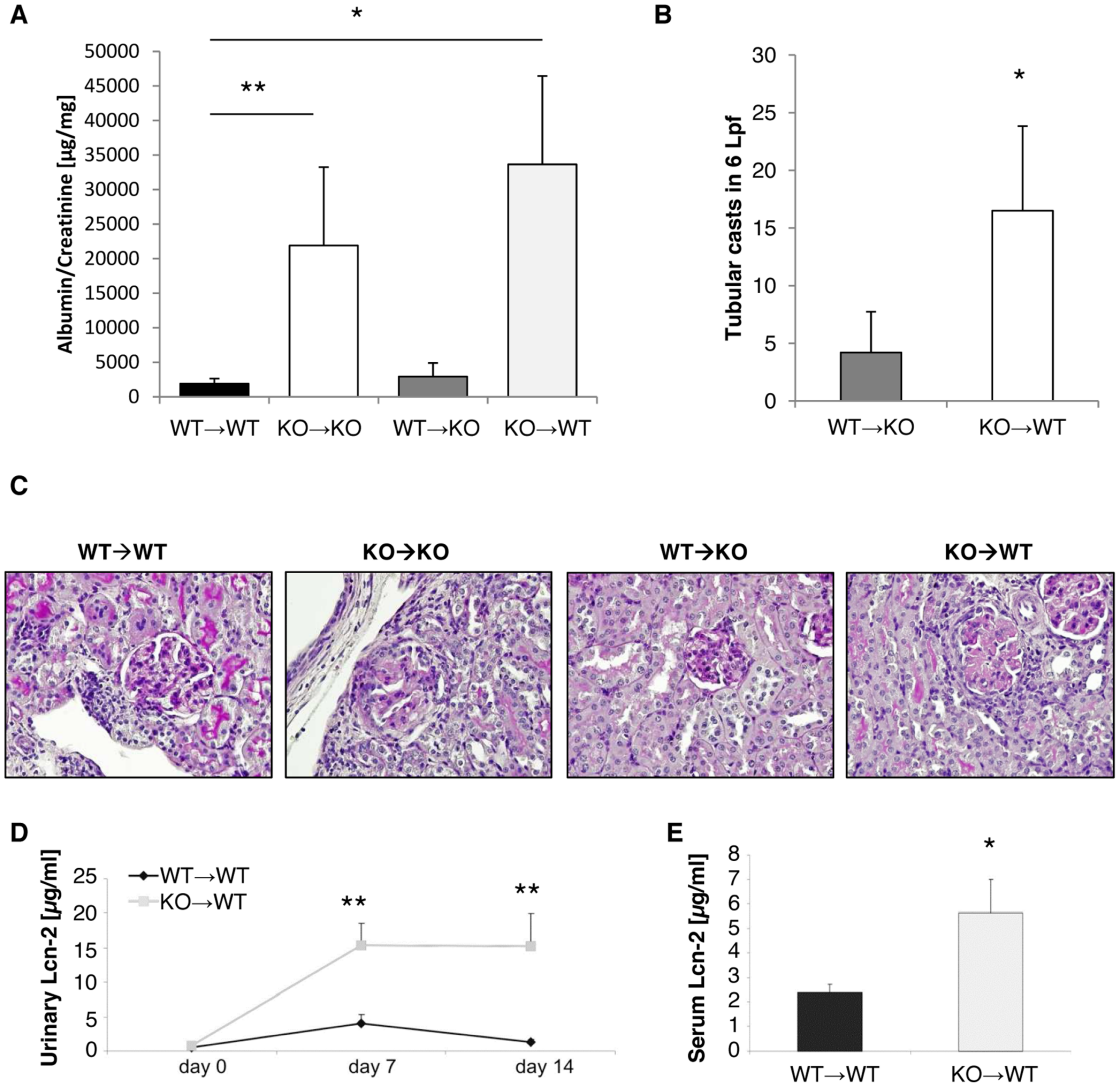
Lcn-2 expressed in circulating immune cells protects mice from NTS. WT (black bar; n = 10), Lcn-2 KO (white bar; n = 8) and chimeras (WT→KO, dark grey bar, n = 6; KO→WT, light grey bar, n = 8) were subjected to NTS. (A) On day 14 after induction of NTS albuminuria was evaluated. (B) Representative PAS-stained kidney sections are shown. Magnfication x400. (C) Tubular necrosis was assessed in WT→KO and KO→WT chimeras by H&E staining. (D) WT mice and KO→WT chimeras were analyzed for their urinary Lcn-2 concentration 0, 7 and 14 days after NTS induction. (E) On day 14 the serum Lcn-2 concentration of the two groups was evaluated. Data are given as mean ± SEM. *p<0.05, **p<0.01.

Since Lcn-2 is expressed not only in tubular epithelial cells, but also in PMN and macrophages, we evaluated their pathogenic role in NTS by inducing NTS in Lcn-2 chimeras. Chimerism of mice was 100% evaluated by real-time PCR of Lcn-2 in circulating peripheral white blood cells (Figure S4). Since kidney injury after NTS induction is known to be significantly less in bone marrow transplanted mice [21], mice were followed in these experiments for an observation period of 14 days. WT mice reconstituted with WT bone marrow cells (WT→WT) after sublethal radiation displayed significantly less albuminuria and histological changes as compared to Lcn-2 KO mice reconstituted with Lcn-2 KO bone marrow cells (Lcn-2 KO→Lcn-2 KO) 14 days after NTS induction. Lcn-2 KO mice reconstituted with WT bone marrow cells (WT→Lcn-2 KO) displayed a phenotype comparable to WT→WT, whereas WT mice reconstituted with Lcn-2 KO bone marrow cells (Lcn-2 KO→WT) displayed increased susceptibility to NTS comparable to Lcn-2 KO→Lcn-2 KO (Figure 6A,C). In parallel to data in Lcn-2 KO vs. WT, Lcn-2 KO→WT displayed significantly increased necrosis compared to WT→Lcn-2 KO as detected by H&E staining (Figure 6B). Interestingly, Lcn-2 KO→WT displayed significantly increased Lcn-2 levels in the urine 7 and 14 days after NTS induction as compared to WT→WT mice (Figure 6D). This was accompanied by increased Lcn-2 serum levels 14 days after NTS (Figure 6E).

### Toll-like Receptor 2 Activation Induces Pro-inflammatory Cytokine Formation but also Lcn-2 Secretion in a Tubular Epithelial and a Macrophage Cell Line

**Figure 7:**
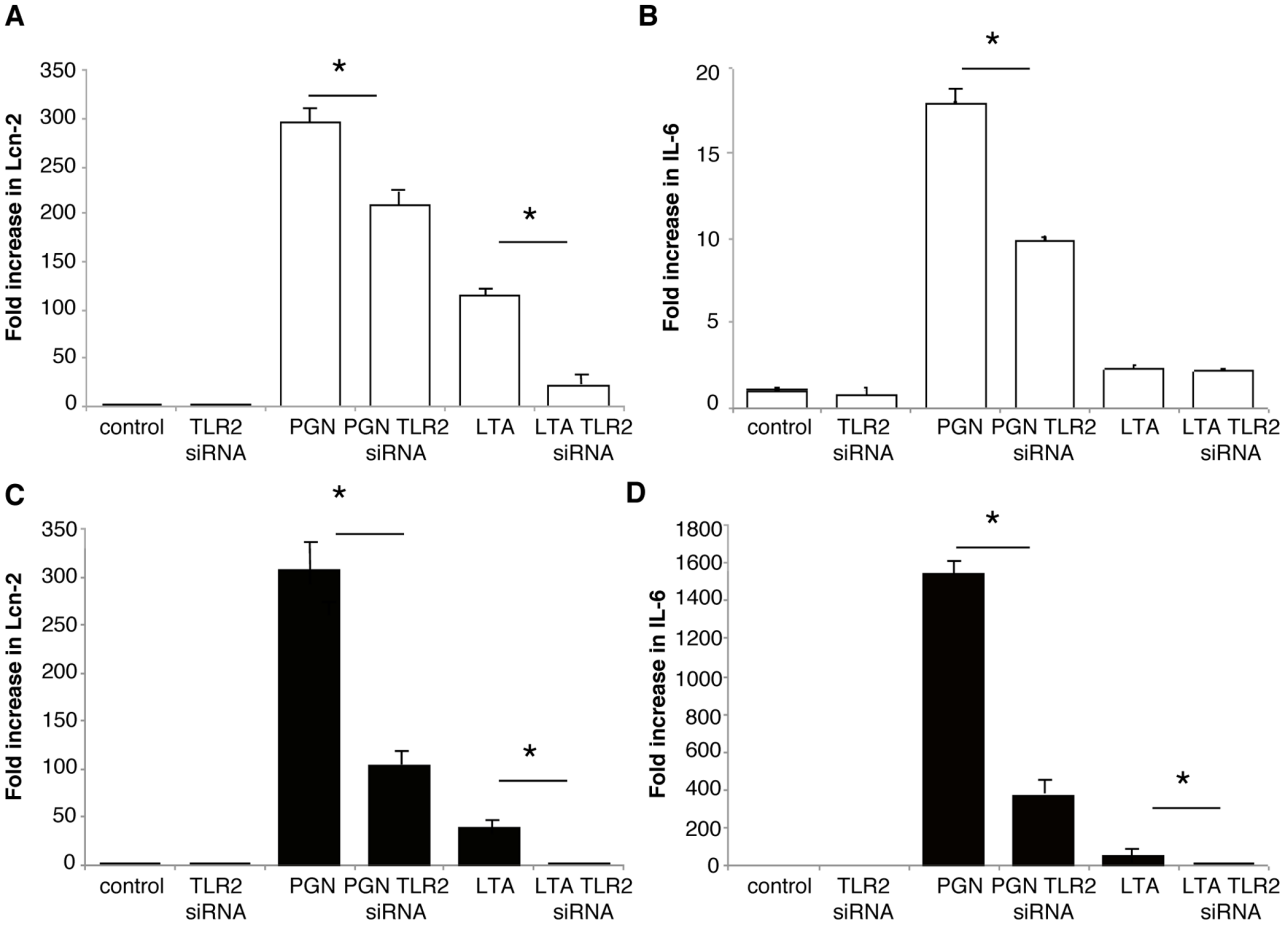
TLR2 stimulation mediates Lcn-2 expression. (A+B) Cultured distal convoluted tubular cells (DCT, white bar) and (C+D) a macrophage cell line (RAW, black bar) were pre-treated with TLR2 siRNA and evaluated for (A+C) Lcn-2 and (B+D) IL-6 mRNA production after stimulation with TLR2 ligands PGN and LTA. All data are provided as fold increase. *p<0.05, **p<0.01. At least three independent experiments were performed.

Since Lcn-2 synthesis can be influenced by Toll-like receptor (TLR) activation [32], we evaluated whether Lcn-2 KO mice show differences in the TLR expression compared to WT mice. Using quantitative PCR analysis we could show that out of all TLRs evaluated (i.e. TLR1–9; Figure S5) only TLR2 and TLR3 were significantly increased in the kidneys of Lcn-2 KO mice compared to WT controls 7 days after NTS induction. Since TLR2 mRNA revealed the most pronounced changes in renal expression (Figure S5), we further focused on the potential role of TLR2 in our system. Similar to the Lcn-2 expression TLR2 was predominantly expressed in tubular and innate immune cells (Figure S6). To prove the functionality we used murine immortalized tubular cells (DCT) and a macrophage cell line (RAW 264.7). TLR2 siRNA reduced TLR2 expression by more than 80% in both cell types (Figure S7). After stimulation with the TLR2 ligands Peptidoglycan (PGN) and lipoteichoic acid (LTA), Lcn-2 expression was significantly up-regulated (up to 300 fold) in both cell lines (Figure 7A+C). Pre-treatment of cells with TLR2 specific siRNA showed a significantly reduced Lcn-2 transcription demonstrating the specificity of TLR2 (Figure 7A+C). Since kidneys of Lcn-2 KO mice showed different cytokine expression patterns compared to WT mice, we also investigated if TLR2 stimulation influences the expression of IL-6. Similar to Lcn-2 induction, PGN and LTA significantly increased the expression of IL-6 up to 18 fold in distal tubular cells (Figure 7B) and up to 1500-fold in macrophages (Figure 7D). Pre-treatment with TLR2-specific siRNA reduced IL-6 expression after stimulation with PGN in both cell lines (Figure 7B+D).

### HMGB-1 Blockade Limits Renal Inflammation and Disease Activity in NTS

**Figure 8:**
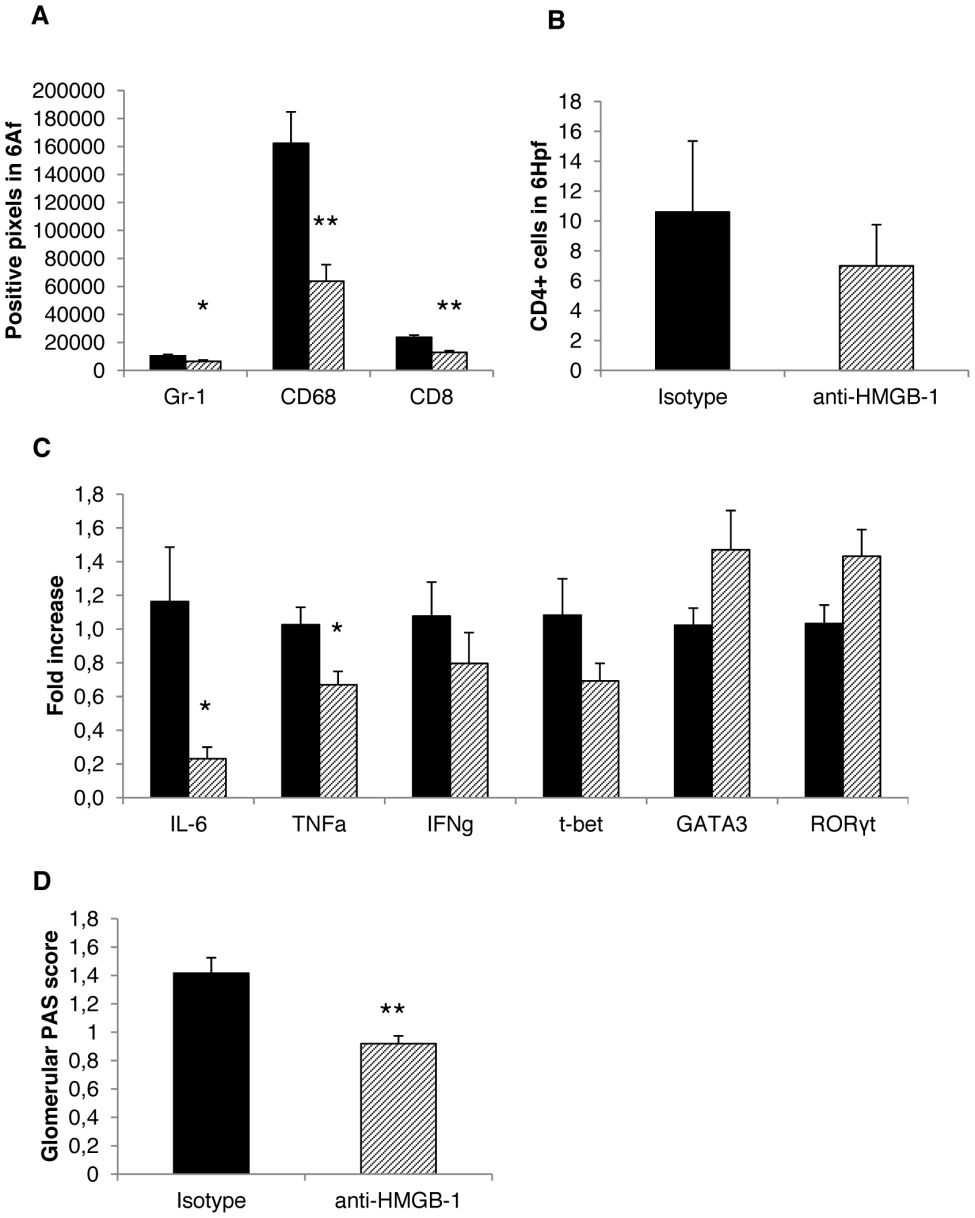
HMGB-1 blockade reduces NTS activity by limiting macrophage infiltration of the kidney of WT mice. WT mice were treated with an isotype control antibody (black bar; n = 5) or with an anti-HMGB-1 antibody (shaded bar; n = 5). Seven days after disease induction (A) staining for Gr-1^+^ cells, CD68^+^ and CD8^+^ cells was performed. The number of positive pixels in 6 analysis fields is given. (B) Staining for CD4^+^ T cells was performed. The number of positive cells in 6 high power fields is given. (C) cDNA isolated from kidney samples was analysed for the expression of the respective genes. The fold change compared to WT with isotype control is shown. (D) Scoring for PAS-positive deposits was performed. All data are given as mean ± SEM. Hpf = high power field, Af = analysis field. *p<0.05 and **p<0.01.

**Figure 9:**
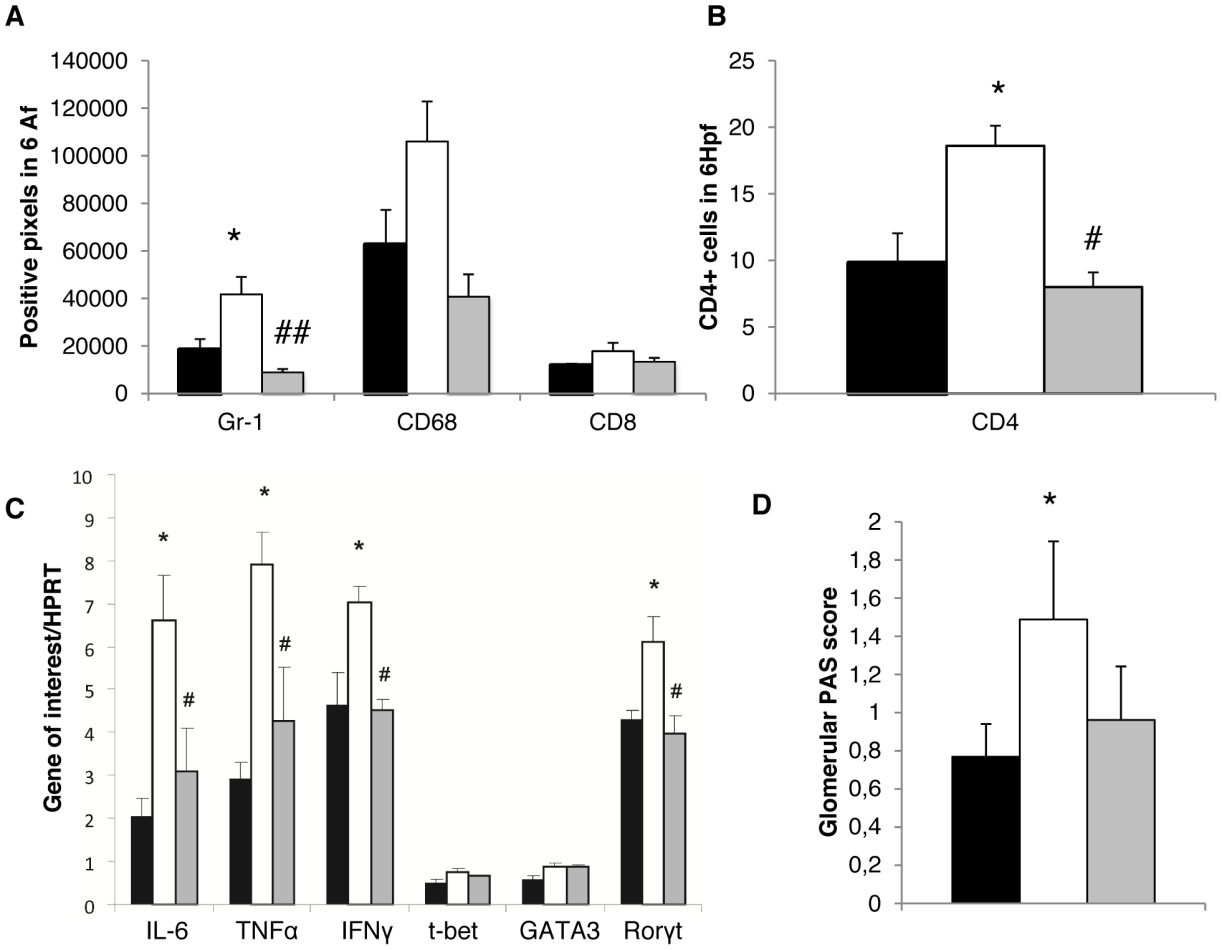
HMGB-1 blockade reduces NTS activity by limiting macrophage infiltration of the kidney of Lcn-2 KO mice. WT mice (black bar; n = 5), Lcn-2 KO mice treated with an isotype control antibody (white bar; n = 6) or with an anti-HMGB-1 antibody (grey bar; n = 5). Seven days after disease induction (A) staining for Gr-1^+^ cells, CD68^+^ and CD8^+^ cells was performed. The number of positive pixels in 6 analysis fields is given. (B) Kidney sections were stained for infiltration by CD4^+^ T cells. The number of positive cells in 6 high power fields is given. (C) cDNA isolated from kidney samples was analysed for the expression of the respective genes. The ratio of the respective gene to the housekeeping gene HPRT is provided. (D) Scoring for PAS-positive deposits was performed. All data are given as mean ± SEM. Af = analysis field; Hpf = high power field; *p<0.05 and **p<0.01 WT compared to Lcn-2KO mice+isotype. ^#^p<0.05 and ^##^p<0.01 Lcn-2KO mice+isotype compared to Lcn-2KO mice+a-HMGB-1 Ab.

To further evaluate the role of increased HMGB-1 that activates the innate immune system via Toll-like receptor (TLR)-2 [30], WT and Lcn-2 KO mice were treated with a HMGB-1 blocking antibody. Independently, both WT and Lcn-2 KO mice displayed significantly decreased numbers of Gr1^+^ PMN in the kidney when treated with a HMGB-1 blocking antibody as compared to isotype control treated mice (Figure 8A, Figure 9A). The same also held true for the numbers of CD68^+^ macrophages, CD4^+^ and CD8^+^ T cells (Figure 8A+B, Figure 9A+B). WT mice treated with anti-HMGB-1 antibody displayed only significant decreases in IL-6 and TNF-α, whereas IFN-γ and Tbet were decreased, but significance was not reached (Figure 8C). RORγt and GATA-3 were increased in kidney of HMGB-1 antibody treated WT mice compared to Isotype-treated WT mice (Figure 8C). In contrast, the cytokines TNF-α, IL-6 and IFN-γ as well as the TH17 master regulator RORγt were found to be significantly decreased in Lcn-2 KO mice treated with the HMGB-1 antibody as compared to isotype-treated Lcn-2 KO mice (Figure 9C). No differences in the mRNA expression of t-bet and GATA-3 were detected (Figure 9C). Finally, both Lcn-2 KO and WT mice treated with the HMGB-1 blocking antibody presented with reduced PAS-positive deposits compared to Lcn-2 KO and WT mice treated with an isotype antibody, respectively (Figure 8D, Figure 9D).

## Discussion

Urinary Lcn-2 has been proven to be a sensitive marker for kidney damage in acute and chronic kidney diseases [1], [2]. Nevertheless, the pathophysiological role of Lcn-2 is largely unknown. Here, we provide evidence that Lcn-2 is not only a reliable marker in renal inflammatory disorders such as NTS, but also a key factor in the development. Lcn-2 expressed in PMN and macrophages revealed to be protective in NTS by inducing apoptosis in these cell populations thereby limiting the production of DAMPs such as HMGB-1 leading to a decreased TLR-2 dependent cytokine production in macrophages and tubular epithelial cells. Because Lcn-2 is induced in NTS by a TLR-2 dependent mechanism this reflects an endogenous feedback mechanism protecting the kidney from an overwhelming inflammation driven damage in NTS (Figure S8).

Lcn-2 is a prominent candidate marker for improving the timely diagnosis of acute kidney injury [2]. In addition, it has been implicated that Lcn-2 might also enhance the diagnosis and state of chronic renal failure [1]. Administration of recombinant Lcn-2 in renal ischemia reperfusion injury (IRI) resulted in a marked improvement of acute renal injury [6], [33]. In chronic renal failure, Viau and coworkers recently provided compelling evidence that Lcn-2 plays a key role in the progression of renal failure via EGFR-mediated proliferation using a non-inflammatory polycystic kidney disease models [13]. In contrast, we detected Lcn-2 to be protective in an inflammatory kidney disease model, namely NTS. Lcn-2 KO mice presented with a massively aggravated infiltration of innate and adaptive inflammatory cells which was accompanied by an increased mRNA expression of Th1- and Th17-dependent cytokines.

Since Lcn-2 is expressed not only in tubular epithelial cells but also in innate immune cells such as PMN and macrophages, we evaluated the NTS outcome in Lcn-2 chimeras. Since WT mice reconstituted with Lcn-2 KO bone marrow cells displayed disease indices comparable to Lcn-2 KO mice, we concluded that Lcn-2 expression in innate immune cells is responsible for the protection from NTS. Lcn-2 has been implicated to be involved in apoptotic processes by acting pro-apoptotic [10], [11]. In our model of NTS, apoptosis, detected by Caspase-3 and TUNEL-staining, revealed to be significantly decreased in the kidneys of Lcn-2 KO mice compared to WT controls thereby suggesting that Lcn-2 production in innate immune cells is crucial for concerted apoptosis in NTS.

To further evaluate this hypothesis, the effects of both Lcn-2 stimulation and blockade of endogenous, constitutive Lcn-2 expression in cultured murine macrophages were investigated. It could be shown that stimulation of macrophages with recombinant Lcn-2 had a minor but significant pro-apoptotic effect. Furthermore, blocking of macrophage Lcn-2 expression using a Lcn-2 specific si-RNA prior to experimentally induced apoptosis had a marked protective effect at least in vitro.

Concerted apoptosis avoiding late apoptosis and necrosis has been shown to decrease the production of intracellular damage-associated molecular patterns (DAMPs) [30], [34]. In line, Lcn-2 KO mice subjected to NTS with impaired concerted apoptosis and increased necrosis produced increased amounts of the DAMP HMGB-1. Moreover, antibody mediated blockade of HMGB-1 *in vivo* protected Lcn-2 KO mice subjected to NTS by limiting the inflammatory response. HMGB-1 seems to act downstream of Lcn-2 since HMGB-1 blockade in WT mice *in vivo* also protected mice from NTS. HMGB-1 has been shown to bind to toll-like receptor 2 (TLR), which is related to its capacity to bind lipopeptides [30]. TLR-2 signalling via DAMPs leads to activation of NF-κB thereby inducing the transcription of NF-κB dependent genes such as TNF-α and IL-6 [30]. Interestingly, we detected increased TLR-2 mRNA expression in Lcn-2 KO mice subjected to NTS. To further evaluate the role of TLR-2 in our model we used an *in vitro* approach. Down-regulation of TLR-2 decreased the production of pro-inflammatory cytokines such as IL-6 in tubular epithelial cells and macrophages. *In vivo*, these data were reflected by increased pro-inflammatory cytokine levels in kidneys of Lcn-2 KO mice, which were significantly decreased by HMGB-1 *in vivo* blockade. Thus, Lcn-2 expressed in innate immune cells induces apoptosis leading to decreased necrosis and production of DAMPs thereby limiting cytokine production and NTS activity.

Lcn-2 has – as the pro-inflammatory cytokines TNF-α and IL-6 - also been shown to be transcribed in a NF-κB-dependent manner [35]–[37]. We found *in vitro* that TLR-2 signalling leads to Lcn-2 mRNA synthesis in tubular epithelial cells and macrophages. Interestingly, *in vivo*, chimeras expressing Lcn-2 only in tubular epithelial cells displayed significantly increased urinary and serum Lcn-2 levels compared to WT controls. Despite these high systemic Lcn-2 levels such mice present with a disease index comparable to Lcn-2 KO mice. These data provide on the one hand direct evidence that the origin of Lcn-2 detected in the urine in inflammatory kidney diseases is rather from tubular cells than from circulating immune cells. On the other hand, it seems to be of utmost importance that Lcn-2 secreted from tubular epithelial cells does not have the capacity to rescue the phenotype. Rather, Lcn-2 has to be present in innate immune cells to exert its anti-inflammatory capacity in inflammatory kidney disease.

In the past, our group provided compelling evidence that immune regulation in NTS is not restricted to the kidney but rather takes place in the regional draining lymph nodes [16], [26], [27], [38]. Obviously, Lcn-2 is a key factor for ameliorating the immune response locally in the kidney rather than in the draining lymph nodes, since cytokine profiling in the lymph node did not reveal a difference between the two groups. Interestingly, a significant increase in the number of monocytes and PMN was detected in the peripheral blood suggesting that there might be also a systemic increase of these two Lcn-2 expressing cell populations in NTS.

Lcn-2 has been implicated not only in apoptosis, but also in proliferation processes [13], [29]. Lcn-2 induces cell proliferation via EGFR activation in non-inflammatory polycystic kidney disease models [13]. In contrast, we detected increased proliferation in our Lcn-2 KO mice not only in infiltrating inflammatory cells but also in tubular epithelial cells. The increased proliferation of tubular epithelial cells might be mediated by the prominent proteinuria in Lcn-2 KO mice as has been shown very recently [39].

In summary, we provide evidence that Lcn-2 plays a protective role in the pathogenesis of NTS. Lcn-2 expressed by innate immune cells leads to concerted apoptosis of immune cells and blocks necrosis and the release and activation of the DAMP HMGB-1. Since HMGB-1 signalling via TLR-2 induces a pro-inflammatory, tissue-damaging cytokine cascade the TLR-inducible formation of Lcn2 acts as an endogenous anti-inflammatory and tissue-protective mechanisms to decrease DAMP-mediated kidney injury in NTS.

## Supporting Information

Figure S1 **Lcn-2 is expressed in Gr-1^+^ PMN and CD68^+^ macrophages.** WT mice were subjected to NTS and followed for 7 days. Kidney sections were double-stained for Lcn-2 (brown) and (A,B) Gr-1 (black) or (C,D) CD68 (black) demonstrating Lcn2^+^Gr-1^+^ and Lcn-2^+^CD68^+^ cells within the glomerulus (black arrow).
(TIF)

Figure S2 **Mouse anti-rabbit IgG antibody concentrations in the serum.** The mouse anti-rabbit IgG concentrations in the serum were evaluated in WT (black bar, n = 7) and Lcn-2 KO mice (white bar, n = 6) 7 days after NTS induction. No significant differences were detectable between the two groups.
(TIF)

Figure S3 **Apoptosis is reduced in Lcn-2 KO mice as early as day 1 and day 4 after NTS induction.** WT (black bar) and Lcn-2 KO (white bar) mice were subjected to NTS and followed for 1 and 4 days (n = 3 per group and time point).
(TIF)

Figure S5 **Evaluation of renal TLR1–9 mRNA in Lcn-2 KO and WT mice subjected to NTS.** WT (black bar, n = 13) and Lcn-2 KO mice (white bar, n = 12) were subjected to NTS and followed for 7 days. Thereafter their kidneys were evaluated for the mRNA expression of TLR1–9 via real-time PCR. The fold increase as compared to the mean expression of the WT mice is given. *p<0.05, **p<0.01.
(TIF)

Figure S6 **TLR2 and macrophage double-staining in NTS-kidneys.** Double-staining for TLR2 (red signal) and macrophages (black signal) was performed on WT kidneys 7 days after NTS induction. A representative picture of a glomerulum is shown. Macrophages are marked by black arrows. Magnification x400.
(TIF)

Figure S7 **TLR-2 is downregulated in a tubular cell and a macrophage cell line.** Real-time PCR for TLR2 was performed in cultured distal convoluted tubular cells (DCT, white bar) and a macrophage cell line (RAW, black bar) after treatment with TLR2 siRNA. *p<0.05. At least three independent experiments were performed.
(TIF)

Figure S4 **Chimerism of mice in circulating peripheral white blood cells.** Three days before induction of NTS, 150 µl of blood was drawn by retroorbital puncture. Peripheral white blood cells were obtained by Histopaque 1083 gradient centrifugation. Afterwards expression of Lcn-2 was analysed by PCR. (+/+ wild type control, +/− heterozygote control; black bar, n = 13; black bar, n = 12)
(TIF)

Figure S8 **Mechanism of Lcn-2 mediated protection of NTS.** Lcn-2 protects macrophages and neutrophils from uncontrolled necrosis by inducing concerted apoptosis. If they lack Lcn-2 they undergo necrosis and HMGB-1 is released. HMGB-1 binds to TLR-2 leading to the production of inflammatory mediators, but also Lcn-2 in innate immune and tubular cells.
(TIF)
