# Supplementary figures and images for: Lipocalin-2 Expressed in Innate Immune Cells Is an Endogenous Inhibitor of Inflammation in Murine Nephrotoxic Serum Nephritis
Source: PLoS One. 2013 Jul 4;8(7):e67693. doi: 10.1371/journal.pone.0067693 (PMC3701542; doi:10.1371/journal.pone.0067693)

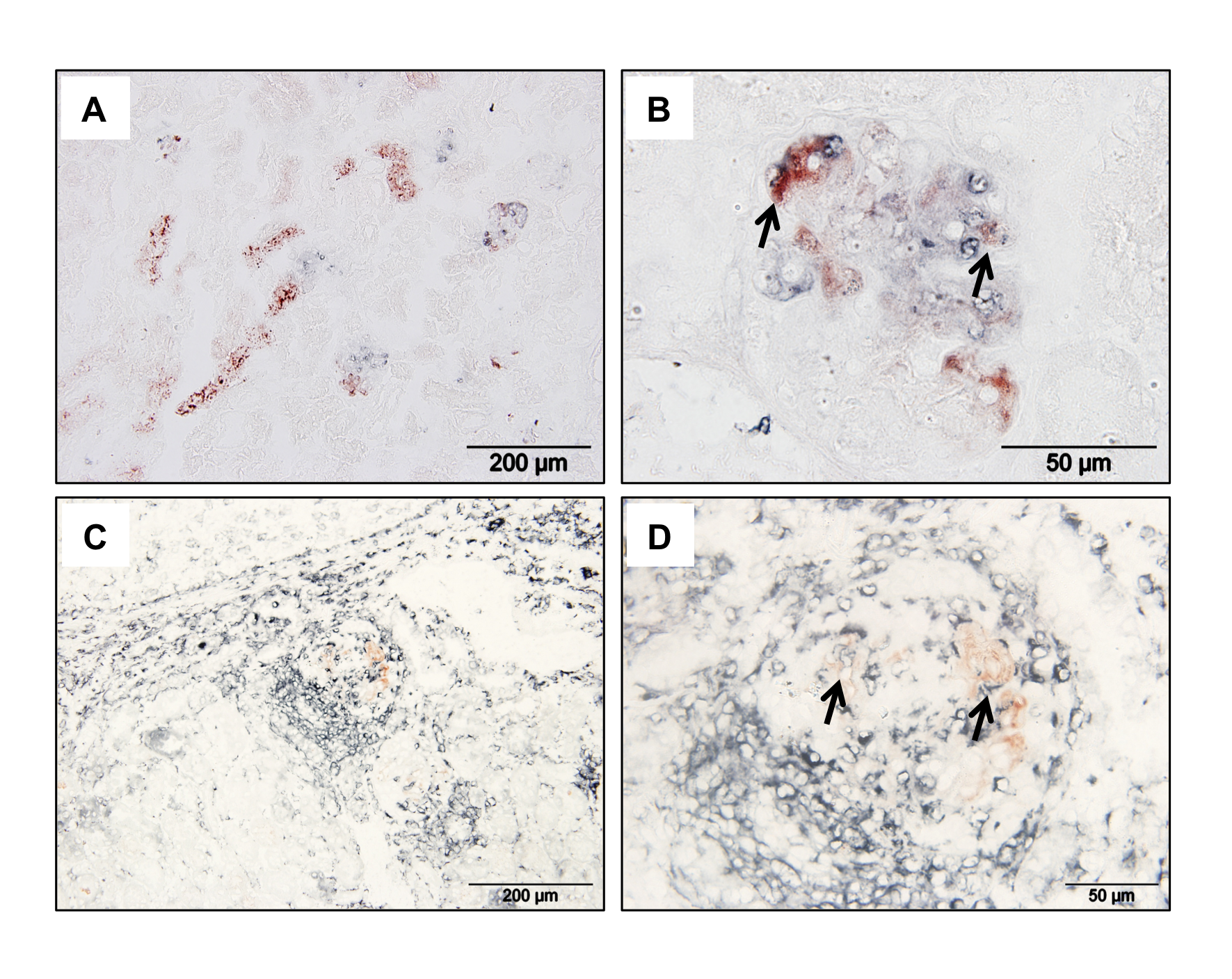

Supplement: Figure S1 — Lcn-2 is expressed in Gr-1+ PMN and CD68+ macrophages. WT mice were subjected to NTS and followed for 7 days. Kidney sections were double-stained for Lcn-2 (brown) and (A,B) Gr-1 (black) or (C,D) CD68 (black) demonstrating Lcn2+Gr-1+ and Lcn-2+CD68+ cells within the glomerulus (black arrow). (TIF) [file pone.0067693.s001.tif]

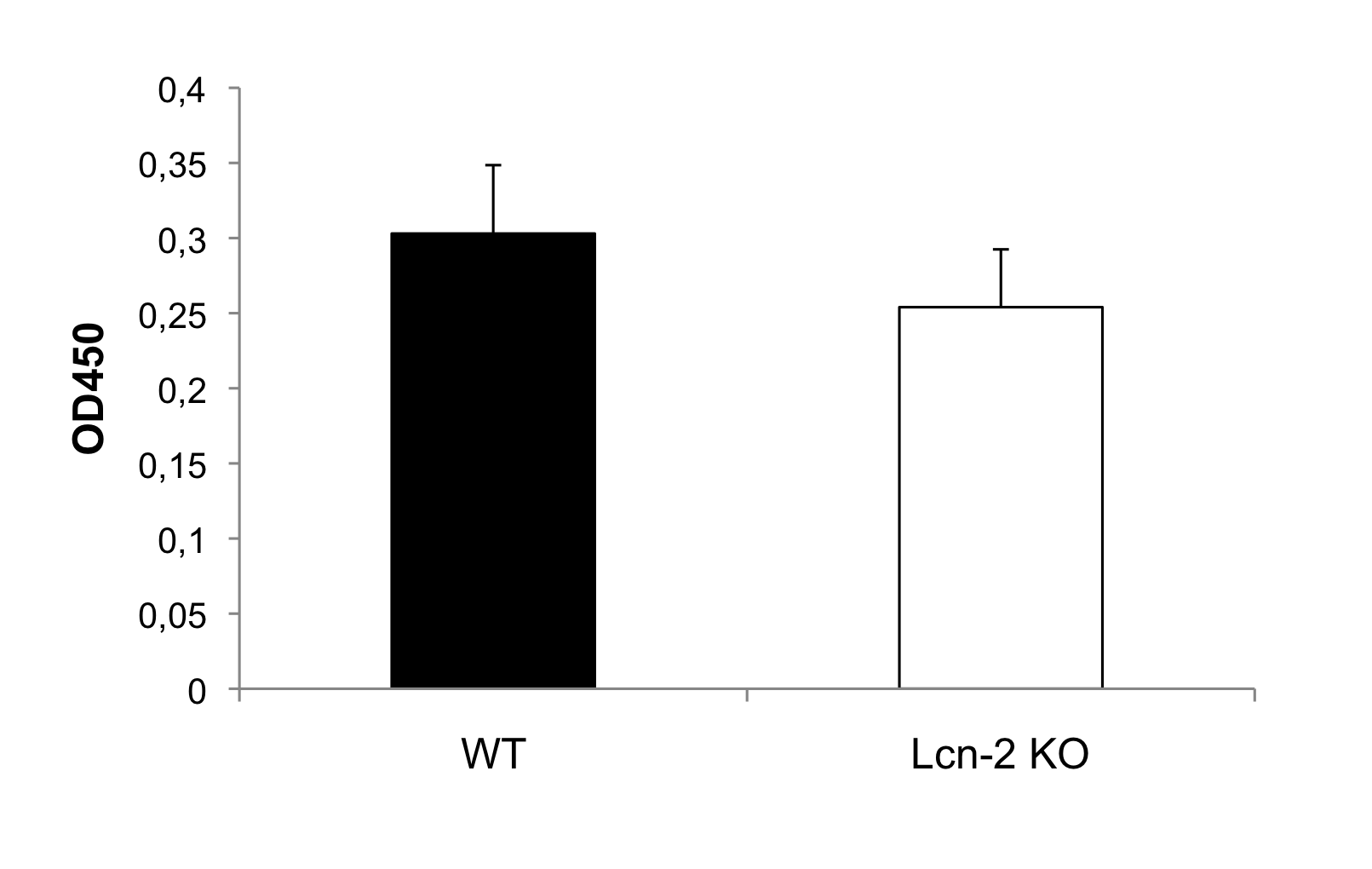

Supplement: Figure S2 — Mouse anti-rabbit IgG antibody concentrations in the serum. The mouse anti-rabbit IgG concentrations in the serum were evaluated in WT (black bar, n = 7) and Lcn-2 KO mice (white bar, n = 6) 7 days after NTS induction. No significant differences were detectable between the two groups. (TIF) [file pone.0067693.s002.tif]

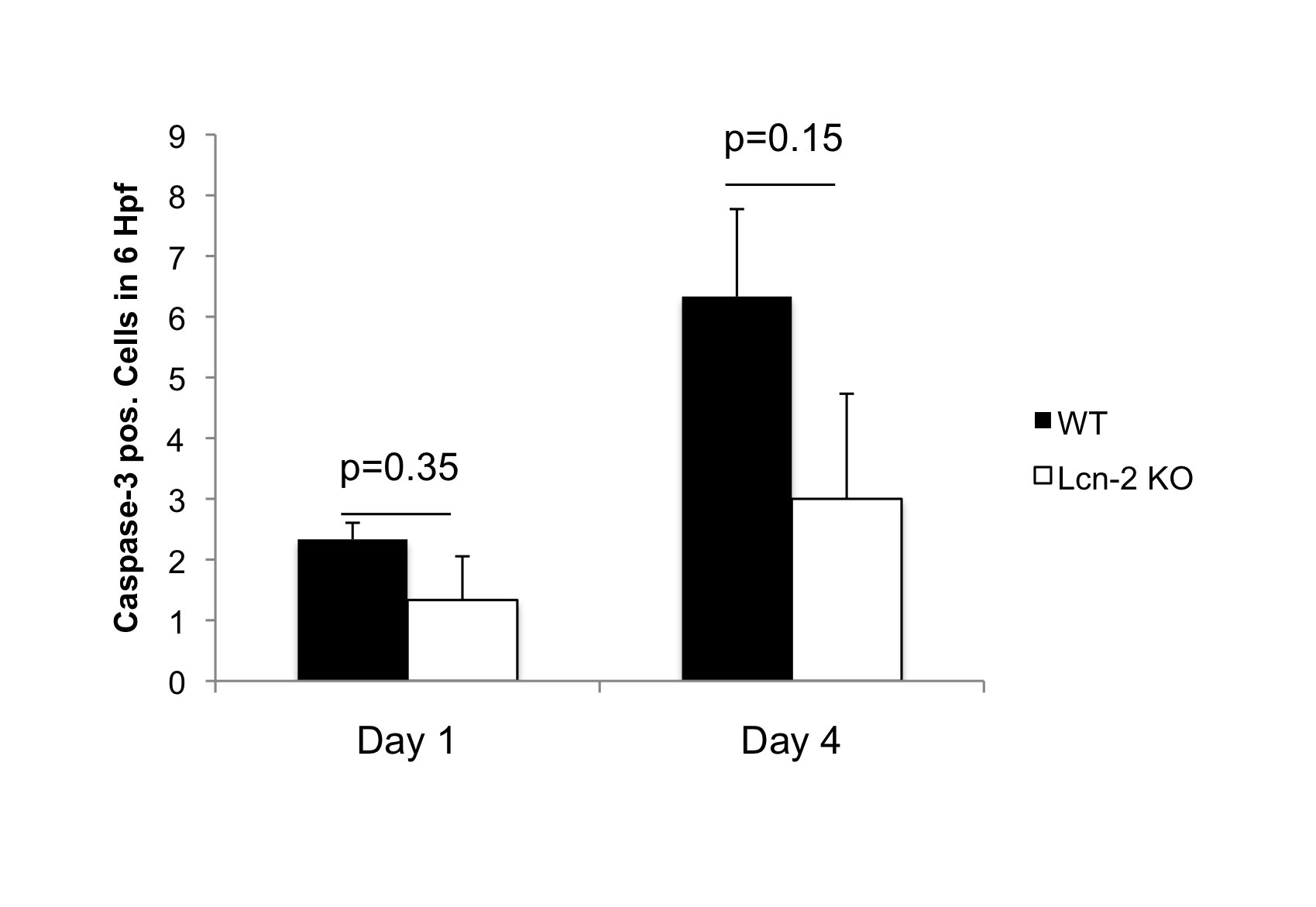

Supplement: Figure S3 — Apoptosis is reduced in Lcn-2 KO mice as early as day 1 and day 4 after NTS induction. WT (black bar) and Lcn-2 KO (white bar) mice were subjected to NTS and followed for 1 and 4 days (n = 3 per group and time point). (TIF) [file pone.0067693.s003.tif]

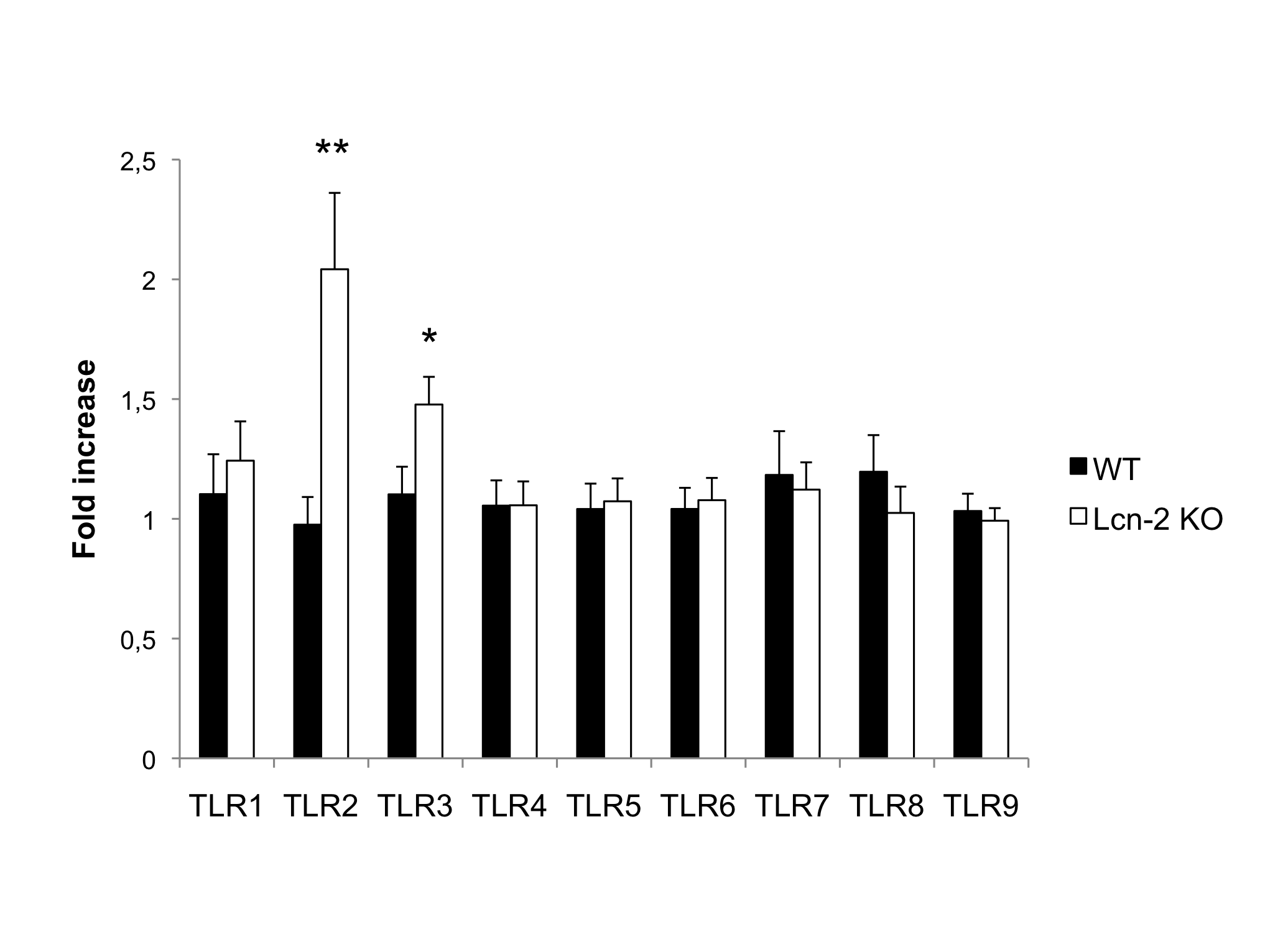

Supplement: Figure S5 — Evaluation of renal TLR1–9 mRNA in Lcn-2 KO and WT mice subjected to NTS. WT (black bar, n = 13) and Lcn-2 KO mice (white bar, n = 12) were subjected to NTS and followed for 7 days. Thereafter their kidneys were evaluated for the mRNA expression of TLR1–9 via real-time PCR. The fold increase as compared to the mean expression of the WT mice is given. *p<0.05, **p<0.01. (TIF) [file pone.0067693.s004.tif]

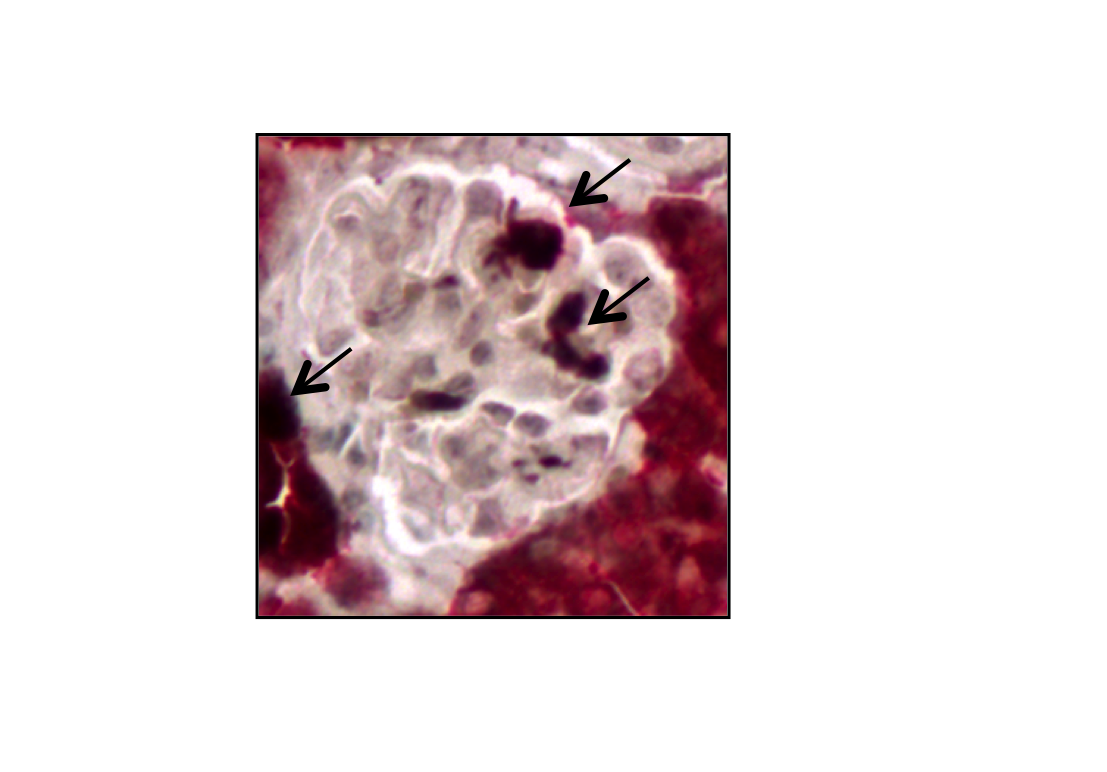

Supplement: Figure S6 — TLR2 and macrophage double-staining in NTS-kidneys. Double-staining for TLR2 (red signal) and macrophages (black signal) was performed on WT kidneys 7 days after NTS induction. A representative picture of a glomerulum is shown. Macrophages are marked by black arrows. Magnification x400. (TIF) [file pone.0067693.s005.tif]

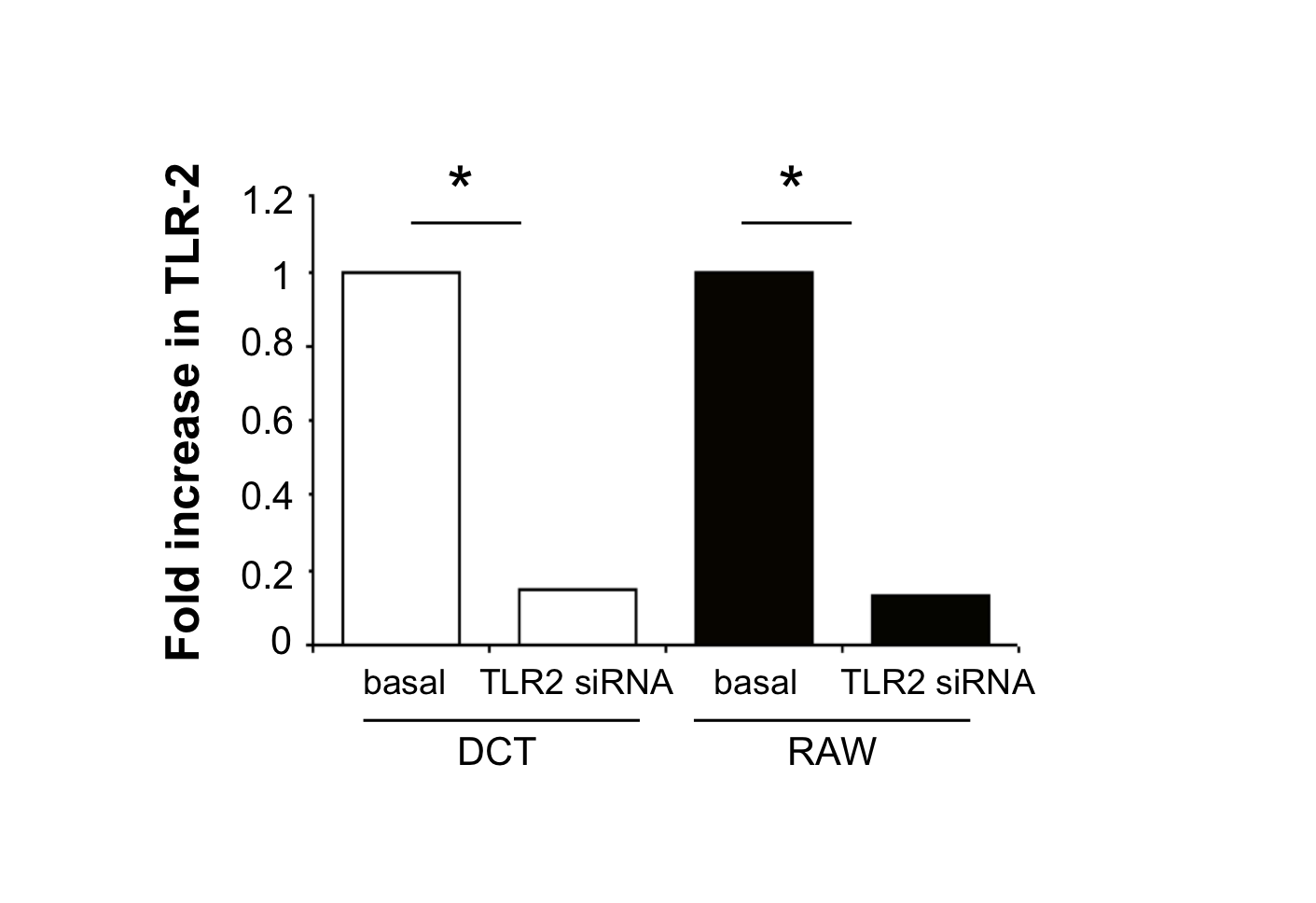

Supplement: Figure S7 — TLR-2 is downregulated in a tubular cell and a macrophage cell line. Real-time PCR for TLR2 was performed in cultured distal convoluted tubular cells (DCT, white bar) and a macrophage cell line (RAW, black bar) after treatment with TLR2 siRNA. *p<0.05. At least three independent experiments were performed. (TIF) [file pone.0067693.s006.tif]

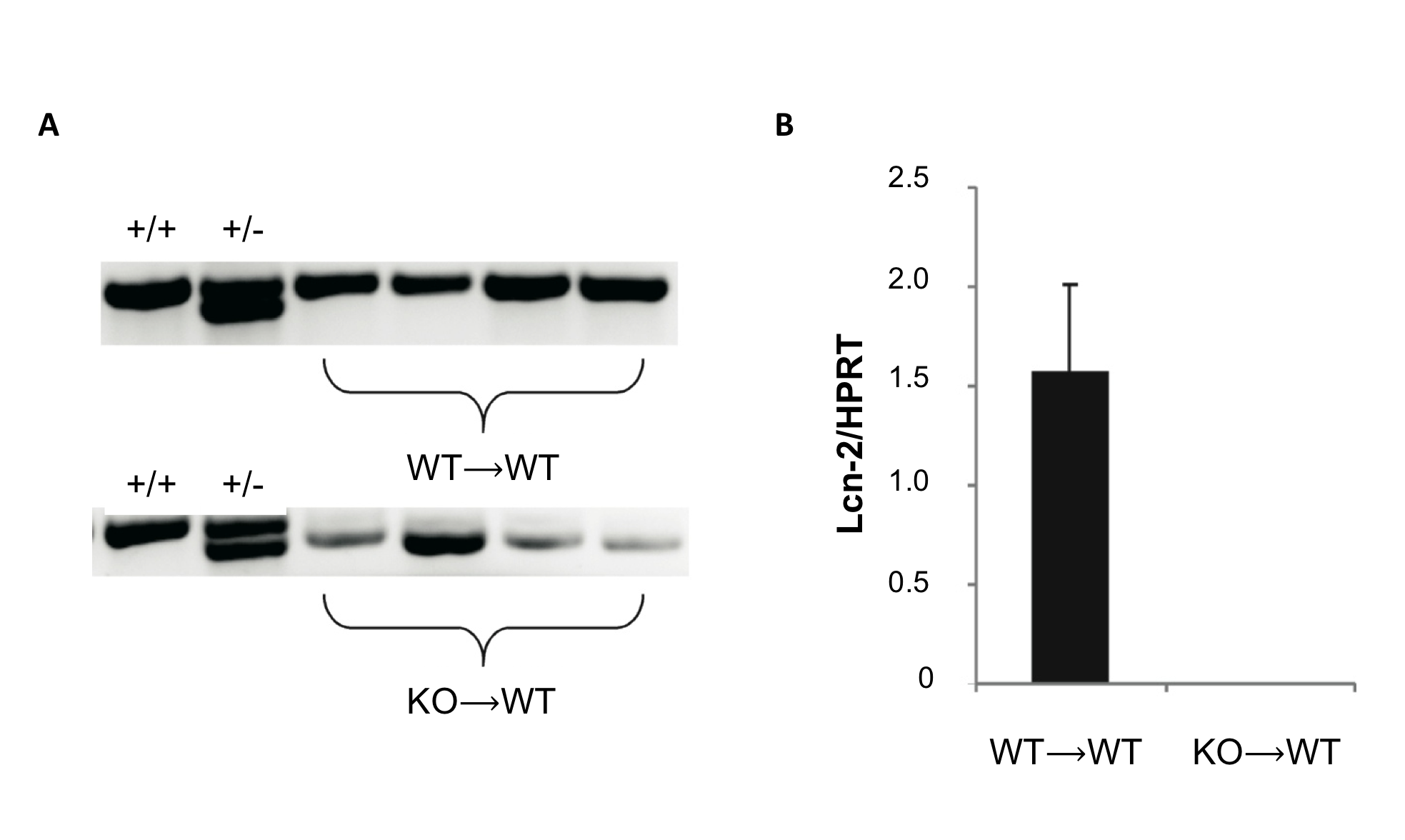

Supplement: Figure S4 — Chimerism of mice in circulating peripheral white blood cells. Three days before induction of NTS, 150 µl of blood was drawn by retroorbital puncture. Peripheral white blood cells were obtained by Histopaque 1083 gradient centrifugation. Afterwards expression of Lcn-2 was analysed by PCR. (+/+ wild type control, +/− heterozygote control; black bar, n = 13; black bar, n = 12) (TIF) [file pone.0067693.s007.tif]

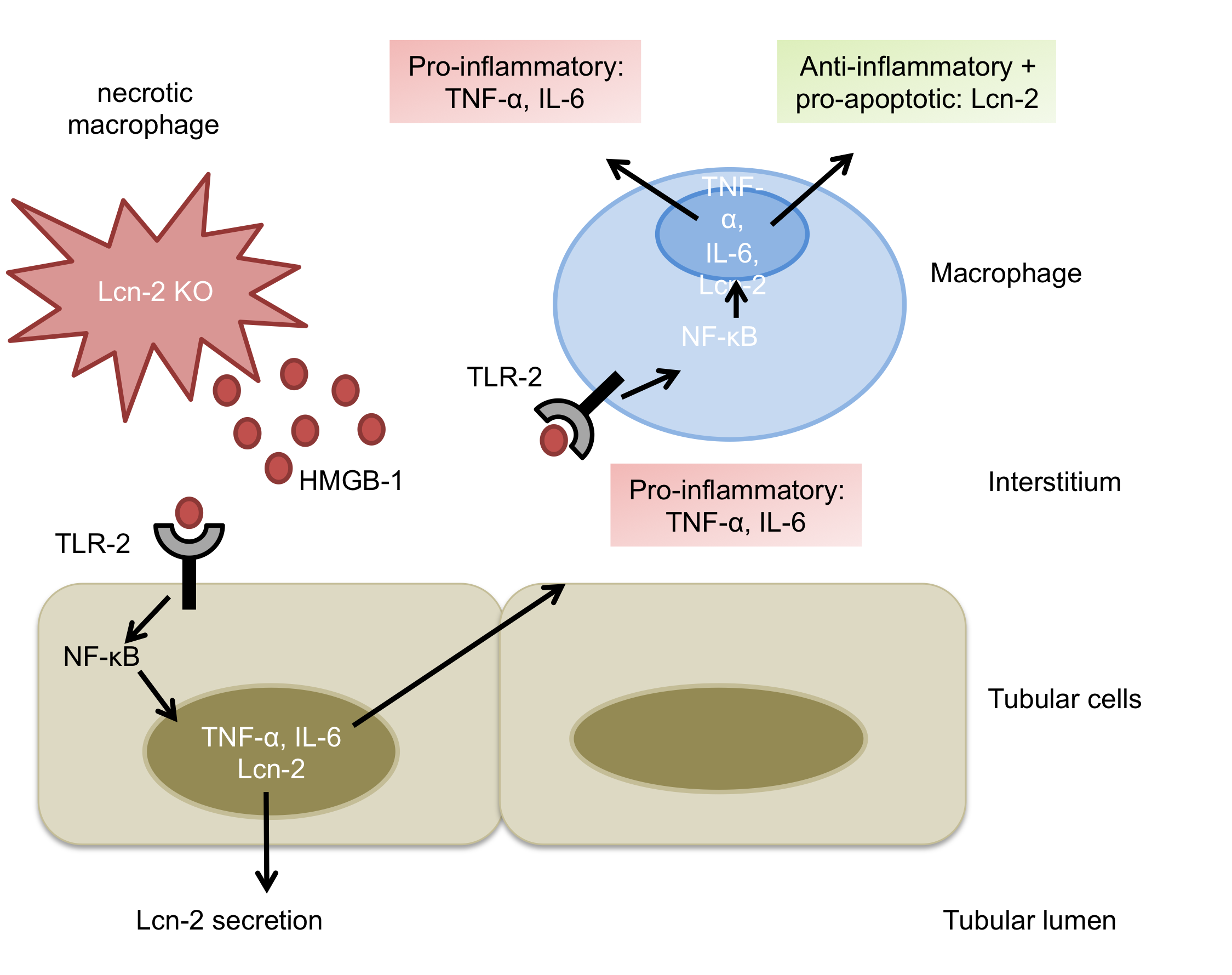

Supplement: Figure S8 — Mechanism of Lcn-2 mediated protection of NTS. Lcn-2 protects macrophages and neutrophils from uncontrolled necrosis by inducing concerted apoptosis. If they lack Lcn-2 they undergo necrosis and HMGB-1 is released. HMGB-1 binds to TLR-2 leading to the production of inflammatory mediators, but also Lcn-2 in innate immune and tubular cells. (TIF) [file pone.0067693.s008.tif]
